# Effectiveness of eHealth Interventions in Improving Treatment Adherence for Adults With Obstructive Sleep Apnea: Meta-Analytic Review

**DOI:** 10.2196/16972

**Published:** 2020-02-18

**Authors:** Jiska Joëlle Aardoom, Lisa Loheide-Niesmann, Hans C Ossebaard, Heleen Riper

**Affiliations:** 1 Department of Clinical, Neuro and Developmental Psychology Vrije Universiteit Amsterdam Netherlands; 2 Department of Public Health and Primary Care Leiden University Medical Center Leiden Netherlands; 3 National eHealth Living Lab Leiden Netherlands; 4 Behavioural Science Institute, Radboud University Nijmegen Netherlands; 5 Dutch National Healthcare Institute Diemen Netherlands; 6 Department of Medical Informatics Amsterdam University Medical Centers Amsterdam Netherlands; 7 Department of Research and Innovation GGZ inGeest Specialized Mental Health Care Amsterdam Netherlands; 8 Amsterdam University Medical Centers, Vrije Universiteit Amsterdam, Psychiatry Amsterdam Public Health Research Institute Amsterdam Netherlands

**Keywords:** obstructive sleep apnea, continuous positive airway pressure, treatment adherence, patient adherence, telemedicine, eHealth, meta-analysis, systematic review

## Abstract

**Background:**

Poor adherence to continuous positive airway pressure (CPAP) treatment by adults with obstructive sleep apnea (OSA) is a common issue. Strategies delivered by means of information and communication technologies (ie, eHealth) can address treatment adherence through patient education, real-time monitoring of apnea symptoms and CPAP adherence in daily life, self-management, and early identification and subsequent intervention when device or treatment problems arise. However, the effectiveness of available eHealth technologies in improving CPAP adherence has not yet been systematically studied.

**Objective:**

This meta-analytic review was designed to investigate the effectiveness of a broad range of eHealth interventions in improving CPAP treatment adherence.

**Methods:**

We conducted a systematic literature search of the databases of Cochrane Library, PsycINFO, PubMed, and Embase to identify relevant randomized controlled trials in adult OSA populations. The risk of bias in included studies was examined using seven items of the Cochrane Collaboration risk-of-bias tool. The meta-analysis was conducted with comprehensive meta-analysis software that computed differences in mean postintervention adherence (MD), which was defined as the average number of nightly hours of CPAP use.

**Results:**

The meta-analysis ultimately included 18 studies (N=5429 adults with OSA) comprising 22 comparisons between experimental and control conditions. Postintervention data were assessed at 1 to 6 months after baseline, depending on the length of the experimental intervention. eHealth interventions increased the average nightly use of CPAP in hours as compared with care as usual (MD=0.54, 95% CI 0.29-0.79). Subgroup analyses did not reveal significant differences in effects between studies that used eHealth as an add-on or as a replacement to care as usual (*P*=.95), between studies that assessed stand-alone eHealth and blended strategies combining eHealth with face-to-face care (*P*=.23), or between studies of fully automated interventions and guided eHealth interventions (*P*=.83). Evidence for the long-term follow-up effectiveness of eHealth adherence interventions remains undecided owing to a scarcity of available studies and their mixed results.

**Conclusions:**

eHealth interventions for adults with OSA can improve adherence to CPAP in the initial months after the start of treatment, increasing the mean nightly duration of use by about half an hour. Uncertainty still exists regarding the timing, duration, intensity, and specific types of eHealth interventions that could be most effectively implemented by health care providers.

## Introduction

Obstructive sleep apnea (OSA) is a clinical sleep disorder characterized by recurrent episodes of partial or complete obstruction of the respiratory passages during sleep [1,2]. Symptoms include choking or gasping during sleep, daytime sleepiness, startled awakening, poor concentration, and difficulty staying asleep [2,3]. The prevalence of OSA in the general adult population has been found to range from 6% to 17% or to be as high as 49% at advanced ages [4]. Continuous positive airway pressure (CPAP) is considered the gold standard for the treatment of patients with moderate-to-severe OSA. It involves wearing a mask during sleep that uses a pump to provide a constant flow of air (pressure) to the throat to keep the airway open. Treatment with CPAP is highly effective for normalizing breathing and sleep; it reduces the frequency of respiratory events during sleep, decreases daytime sleepiness, and improves blood pressure and quality of life [5,6].

Unfortunately, acceptance and adherence are often suboptimal in CPAP treatment, thereby jeopardizing the improved health outcomes. It is estimated that 30% to 80% of OSA patients can be classified as nonadherent when operationalized as using CPAP for less than 4 hours per night [7-9]. Numerous factors have been linked to CPAP nonadherence, although no single factor has been consistently identified. Many factors presumably interact and may jointly predict nonadherence [7,10,11], including patient characteristics (eg, age, race, and smoking status) [7,12], disease characteristics (eg, symptom severity) [7,12], experienced side effects (eg, skin irritation, dryness in the nose or mouth, and abdominal bloating) [11], treatment titration procedures [8], and psychosocial factors (eg, skills at coping with challenging situations, mental health problems, self-efficacy, and social support) [7,11,12].

A growing body of research is investigating interventions to promote CPAP adherence [12,13]. Such interventions may incorporate educational, supportive, and therapeutic strategies, such as cognitive-behavioral techniques. A Cochrane review by Wozniak et al [13] reported low- to moderate-quality evidence for these types of adherence interventions. Behavioral interventions were found to have the largest effects on CPAP adherence, followed by supportive interventions and educational interventions. More specifically, the respective intervention strategies yielded mean improvements of 1.5 hours, 50 minutes, and 35 minutes of CPAP use per night.

Strategies delivered by means of information and communication technologies (ie, eHealth) offer strong potential to address the relatively poor rate of CPAP adherence through standardized education, real-time monitoring of symptoms and CPAP adherence in daily life, self-management, and early identification and intervention if device or treatment problems arise [14-17]. With regard to the then existing evidence base on eHealth adherence interventions, Sawyer et al [8] briefly reviewed technological strategies to promote CPAP adherence. They concluded that most strategies were promising in terms of effect sizes but that larger trials were needed to determine their potentials. A similar conclusion was reached in a more recent review, which mainly focused on remote telemonitoring [15]. Overall, preliminary evidence suggests that eHealth technology has the potential to improve patient adherence. To the best of our knowledge, however, no studies have systematically assessed the impact of the broad range of available eHealth technologies on CPAP adherence. This meta-analytic review investigated the effectiveness of eHealth interventions in improving CPAP adherence in adult populations with OSA.

## Methods

### Search Strategy

Our search strategy was part of a broader search performed in a research project on the role of eHealth in treatment adherence in chronic lung diseases. The searches for OSA, asthma, and chronic obstructive pulmonary disease (COPD) were thereby pooled together.

A systematic literature search was conducted in the electronic databases of the Cochrane Library (Wiley), PsycINFO (EBSCO), PubMed, and Embase. The search results were limited to available full-text articles in English or Dutch with publication dates from January 1, 2000, to March 20, 2018. The starting year of 2000 was chosen because technology began greatly advancing around that time. Terms related to eHealth technology, patient adherence, and the target populations were combined, using both free-text and index terms (see Multimedia Appendix 1 for the full search string). We additionally checked reference lists in the ultimately included studies, as well as systematic reviews on the research topic to locate other potentially relevant studies.

### Eligibility Criteria

The study inclusion criteria were as follows: (1) The target population comprised patients aged 18 years or older who were undergoing CPAP treatment and whose OSA diagnosis was supported by polysomnographic examination, home sleep apnea testing, or nocturnal pulse oximetry; (2) A major component of the experimental intervention was delivered by eHealth technology or an eHealth component was assessed as an add-on to care as usual (CAU), irrespective of whether it comprised a major part of the experimental intervention. The criteria to qualify as an eHealth intervention were that the intervention was delivered via information and communication technology, such as telephone calls, telemedicine (eg, videoconferencing), websites, smartphone applications, SMS and the intervention was delivered independently of time and place, making distance a critical factor (eg, videos delivered in face-to-face sessions were not considered eHealth interventions); (3) CAU did not include the experimental eHealth intervention or component under investigation, thus excluding any studies comparing similar eHealth interventions with differing contents, such as general versus tailored text messages; (4) Outcomes were assessed in terms of one or more quantitative measures of patient adherence to CPAP treatment; (5) Outcomes were compared statistically between study conditions; (6) Study design was a randomized controlled trial.

### Screening

Two reviewers (JA and LL) independently screened all titles and abstracts for eligibility. Subsequently, the reviewers independently screened the full text of the selected papers to determine eligibility for inclusion. Disagreements were resolved by discussion. Covidence software [18] was used to manage the screening process and risk-of-bias assessments.

### Data Extraction, Syntheses, and Analyses

Data on study reference, design, population, interventions, outcomes, and results were extracted by JA from all eligible studies (Multimedia Appendix 2). Where feasible, data were synthesized using a narrative approach and a statistical approach (ie, meta-analysis). The meta-analysis was conducted with Comprehensive Meta-Analysis software (CMA, version 3.3.070, Biostat, Englewood, New Jersey), which analyzed the computed differences in means (MD) in adherence measures (the average number of nightly hours of CPAP use). The meta-analysis was performed on available postintervention data.

For studies with multiple intervention conditions, the control condition was split into two or more groups corresponding to the number of experimental comparisons, with sample sizes divided by that number, thus enabling separate comparisons of intervention conditions within the same meta-analysis. Since considerable heterogeneity among studies was expected, a random-effects model was chosen [19]. Heterogeneity between observed effect sizes was examined with the *I^2^* statistic. To calculate 95% CIs around *I^2^*, we used the noncentral χ^2^-based approach within the HETEROGI module for Stata [20]. Funnel plots were visually inspected to assess potential publication bias, and the Duval and Tweedie trim-and-fill procedure [21] was conducted to adjust for any such bias. Additionally, funnel plot symmetry was checked using the Egger linear regression test of the intercept [22]. Statistical outliers were defined as studies in which the 95% CI of the MD did not overlap with that of the pooled MD. If outliers were identified, sensitivity analyses were performed by removing them from the analysis to ascertain whether exclusion would significantly affect the results.

Subgroup analyses were conducted using a mixed-effects model, pooling the studies within subgroups with a random-effects model and testing for significant differences between subgroups with a fixed-effects model. One subgroup analysis compared CPAP adherence in studies that tested eHealth interventions as an add-on to CAU with adherence in studies that tested them as a replacement of CAU. This was of interest because the context of eHealth delivery could have important implications for how interventions are implemented in the process of care delivery and follow-up, and more generally, for the efficiency of and burden on the health care system. A second subgroup analysis compared interventions delivering eHealth only versus blended approaches combining eHealth and face-to-face strategies. A third analysis compared fully automated versus guided eHealth interventions given that it is often assumed that guided and blended interventions lead to better adherence outcomes.

If included studies did not report the data needed to carry out main or subgroup analyses, we attempted to contact the first or corresponding author to gain the necessary data.

### Risk-of-Bias Assessment

The Cochrane Collaboration risk-of-bias tool [23] was used to assess the quality of all included studies. Two reviewers (JA and LL) independently evaluated the following dimensions of the risk of bias: (1) adequacy of random sequence generation; (2) adequacy of concealment of the allocation sequence to personnel; (3) blinding of study participants and personnel; (4) blinding of outcome assessors; (5) adequacy of handling of incomplete outcome data; (6) selective outcome reporting; and (7) potential other sources of bias, such as baseline imbalances and differential dropout. Each study was rated on every dimension as “low risk,” “high risk,” or “unclear risk.” Disagreements were resolved by discussion.

## Results

### Search Results

**Figure 1:**
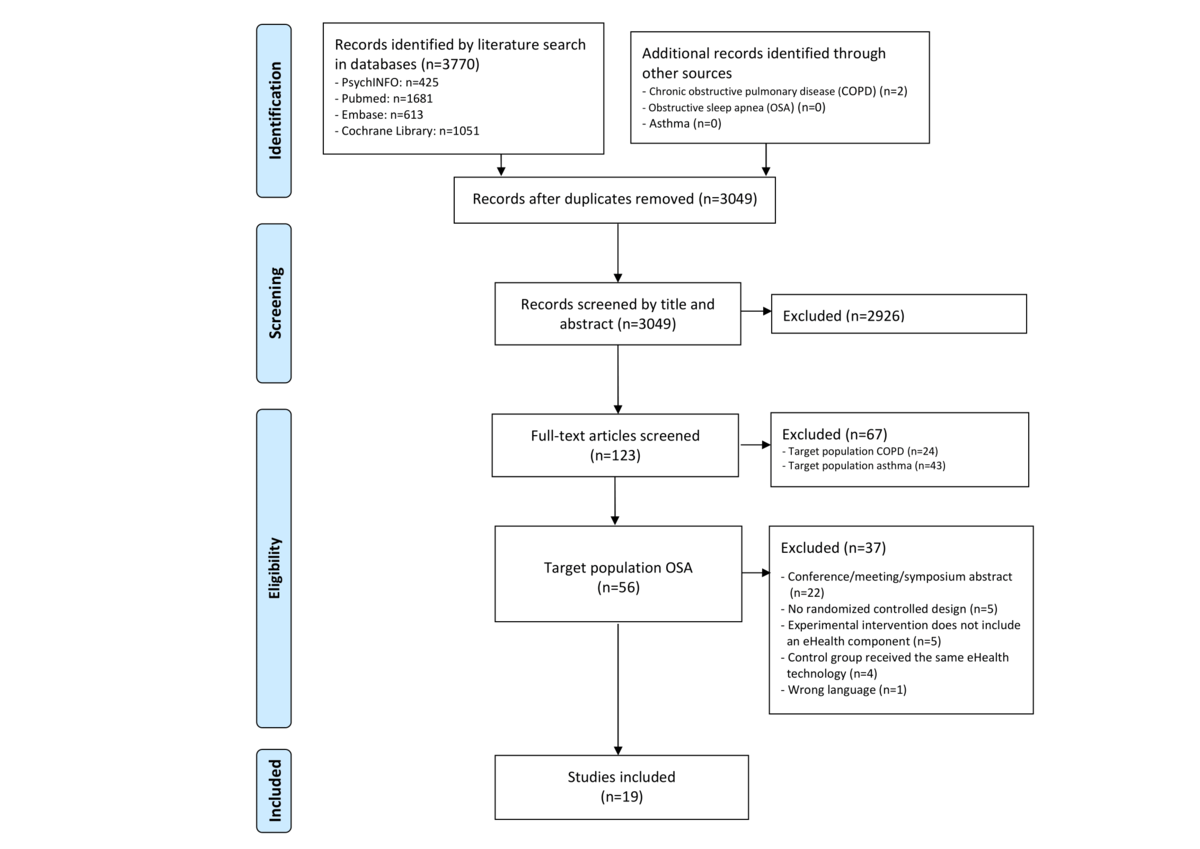
PRISMA flowchart describing the study identification and selection process.

Figure 1 presents the PRISMA flow diagram depicting the process of the literature search, identification, and selection. The pooled systematic search for OSA, asthma, and COPD resulted in a total of 3772 potentially relevant articles. After removal of duplicates (n=723), a total of 3049 articles were selected for title and abstract screening. Subsequently, 123 studies were selected for full-text screening, and 56 of these were found to target OSA. A total of 19 studies targeting individuals with OSA were eventually included in the narrative review, and 18 of these were included in the meta-analysis.

### Study Characteristics

Multimedia Appendix 2 provides an overview of the relevant characteristics of each of the included studies. All studies focused on adults with OSA who were starting either CPAP or automatically adjusted positive airway pressure (APAP) treatment. Adherence to CPAP was assessed mostly in terms of average nightly CPAP use in hours with or without the criterion “on nights being used,” the percentage of nights of CPAP use with or without the criterion “for more than X hours per night,” or the percentage of patients adherent to CPAP.

Most studies (n*=*14) compared CAU with and without supplementation by one or more eHealth components. For reasons of brevity, these are henceforth called add-on studies. In the remaining five studies, the eHealth component or components were used to replace CAU rather than supplement it. These will be referred to as replacement studies.

Of the 14 add-on studies comparing CAU to the same care supplemented with eHealth, nine studies added eHealth components only, whereas five added a combination of face-to-face and eHealth strategies. Most studies adding eHealth components alone used telemonitoring tools (n=7) to monitor CPAP adherence and efficacy data, and telephone calls (n=7) intended to educate, provide support, promote self-management, or reinforce adherence. One study included a Web-based education portal, as well as automated feedback messages by e-mail, telephone, or SMS, according to CPAP monitoring data [24]. Mendelson et al [25] gave study participants a smartphone with an application incorporating a self-monitoring tool capable of transmitting clinical information and providing self-care messages in daily pictograms. In the five studies that added a combination of face-to-face and eHealth strategies, the eHealth component generally consisted of telephone calls designed to troubleshoot, provide support and encouragement, and reinforce CPAP treatment adherence. The face-to-face components mainly involved personal consultation for education, consultation, or early review [26-29], and one included a brief motivational enhancement program [30].

In the five replacement studies, face-to-face follow-up consultations were replaced by eHealth strategies. More specifically, Fields et al [31] replaced four face-to-face follow-up visits by one video-conferencing consultation and three telephone calls. Three other studies replaced face-to-face visits by telemonitoring units and subsequent collaborative management [32] or by “as needed” clinical contact (eg, in response to mask leaks or low adherence) [17,33]. Isetta et al [34] replaced two face-to-face follow-up visits with follow-up care at a distance as follows: two video-conferencing visits, “as needed” televisits or telephone calls, and a Web-based portal including education, self-monitoring, and a messaging tool for communicating with staff to solve treatment-related problems.

All studies, except one [34], included postintervention assessments between 1 and 4 months after baseline. Five studies included follow-up assessments after completion of the intervention [26,30,33,35,36], ranging from 1 month [30] to 2 years [26].

As shown in Multimedia Appendix 2, the types and intensities of CAU varied considerably. Participants typically received education about OSA and CPAP, treatment instructions, and one or more follow-up assessments by sleep practitioners via telephone calls, home visits, or patient visits to the clinic.

### Risk-of-Bias Assessment

**Figure 2:**
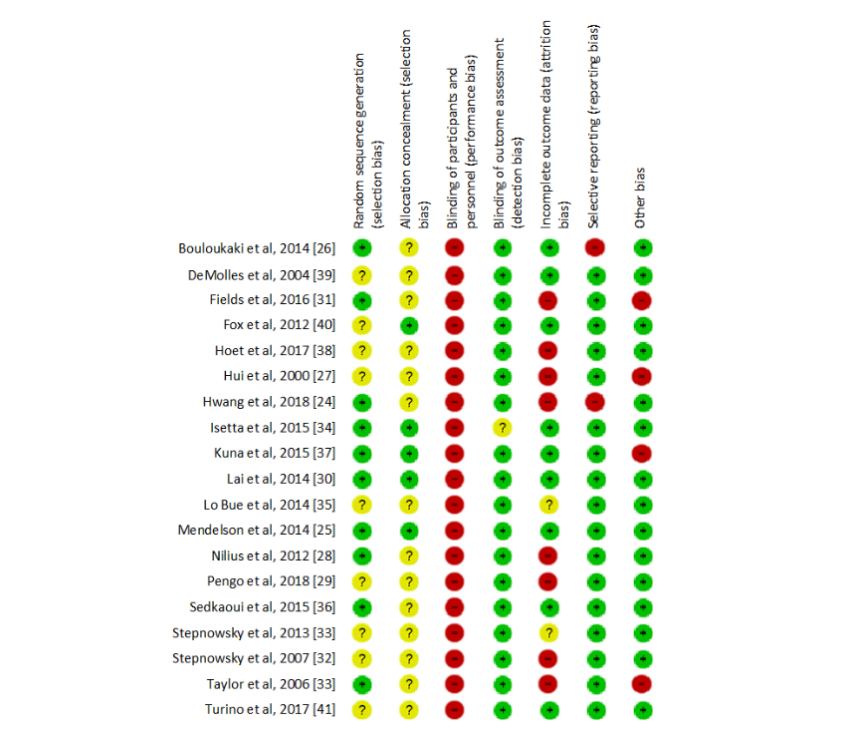
Risk of bias for each individual study included in this meta-analytic review.

**Figure 3:**
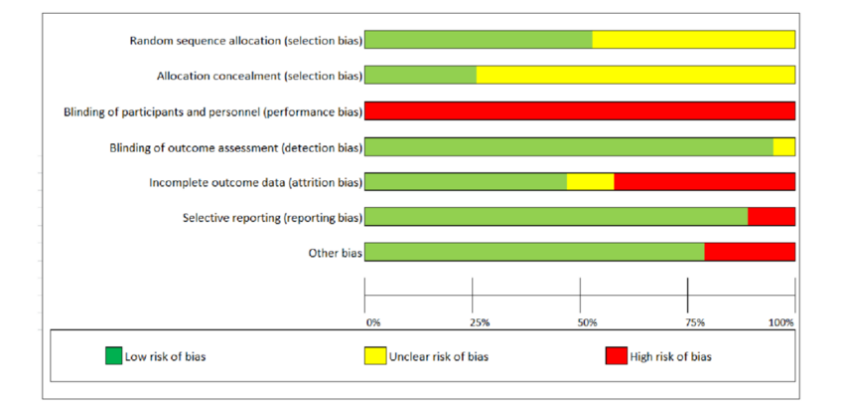
Summary of the risk of bias for all included studies in this meta-analytic review.

Figure 2 presents the results of the risk-of-bias assessment for each study separately, and Figure 3 summarizes the percentages of studies with low, unclear, and high risks of bias. The methodological quality of the studies varied considerably. One study had a low bias risk for only two of the seven risk-of-bias criteria, eight had it for three criteria, four had it for four criteria, another four had it for five criteria, and two had it for six criteria.

Not a single study was rated as having a low bias risk for all seven assessment dimensions, and this was mainly due to a high bias risk for the blinding of participants and personnel dimension. Most studies had a low bias risk for blinding of outcome assessment because CPAP adherence data were downloaded directly from CPAP devices. A high risk of selective outcome reporting was identified for two studies that failed to adequately report on the types of adherence outcomes specified in their methods sections [24,26] or on the outcome periods defined there [26]. Studies with a high risk of attrition bias (Figure 2) generally did not analyze the data according to an intent-to-treat design, thus excluding participants who did not adhere to the intervention or were lost to follow-up. Finally, identified high risks of other sources of bias (n=4) were in two studies related to significant baseline differences (*P*<.05) that were not controlled for in the analyses [27,31]. Another study reported that about 80% of participants receiving CAU or CAU plus Web access to airway pressure data were treated with APAP rather than CPAP, whereas APAP was used in a third study arm by only 62% of participants [37]. In a fourth study, bias may have arisen in the follow-up period because of increased face-to-face walk-in care received by the CAU group, which was balanced with an increased number of telephone contacts in the telemedicine group [17].

### Publication Bias

A visual inspection of the funnel plot did not indicate potential publication bias, but the Egger linear regression test of the intercept was significant (*P*=.02). However, no studies were removed and imputed by the trim-and-fill procedure, suggesting no evidence of publication bias.

### Meta-Analysis of eHealth Interventions and Continuous Positive Airway Pressure Adherence

**Figure 4:**
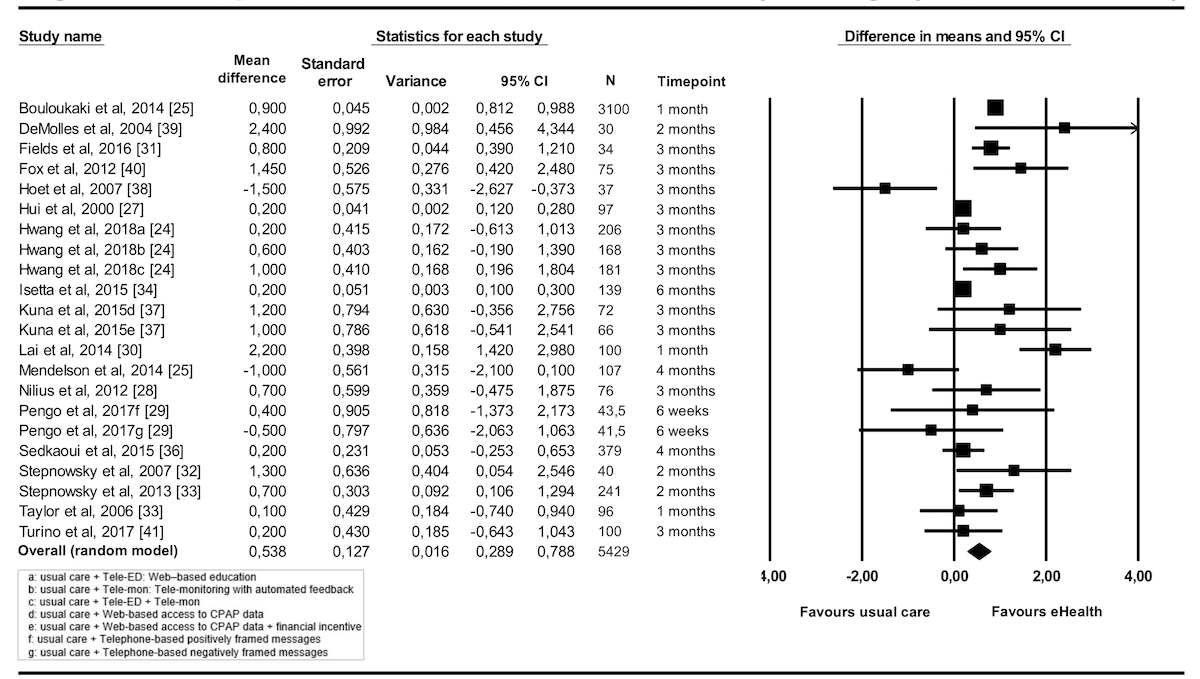
Forest plot of intervention effects on adherence as defined as mean nightly continuous positive airway pressure (CPAP) use in hours.

Among the 19 eligible studies identified, one study [35] had to be excluded from the meta-analysis because postintervention data on average nightly CPAP use was lacking, being provided at a 1-year follow-up only. The results of the remaining 18 studies, which contained 22 comparisons between experimental and control conditions, are shown in Table 1 and Figure 4. The use of eHealth interventions as a supplement or replacement of CAU was associated with a significant improvement in patients’ average nightly CPAP use in hours at the postintervention measurement (MD=0.54, 95% CI 0.29-0.79), with high heterogeneity (*I^2^*=90%, 95% CI 87-93). The exclusion of studies identified as outliers [24-27,30,38] resulted in a similar rounded mean difference (Table 1), with a considerable decrease in heterogeneity (*I^2^*=51%, 95% CI 10-73).

Because one study [35] could not be included in our meta-analysis on postintervention data, we will review its postintervention results narratively. Directly after the intervention period of 1 month, the monthly average number of nights when the CPAP device had been used for 4 or more hours was significantly higher among participants who received CAU plus early extra telephone support and advice than among those who received CAU only (*P*=.02). The extra-support participants also showed a significantly higher rate of adherence, defined as using CPAP for ≥4 hours a night for at least 70% of the nights.

**Table 1:** Results of the main and subgroup analyses at postintervention assessment.

Variables			Studies, n		Comparisons, n		Total, N^a^		Mean difference (95% CI)		*P* value^b^		*I²* (95% CI)
CPAP^c^ adherence^d^			18		22		5429		0.54 (0.29-0.79)^e^		N/A^f^		90.45 (87-93)
Outliers excluded			12		14		1433		0.54 (0.27-0.82)^e^		N/A		51.10 (10-73)
Subgroup analyses													
**Context of experimental care**											.95		
	Add-on to usual care	13		17		4879		0.54 (0.20-0.87)^e^				91.34 (88-94)	
	Replacement of usual care	5		5		550		0.52 (0.13-0.91)^e^				69.10 (21-88)	
**Medium of experimental care**											.23		
	eHealth only	11		14		1690		0.38 (0.07-0.70)^e^				66.35 (41-81)	
	Blended: combined eHealth + face-to-face care	5		6		3458		0.76 (0.23-1.29)^e^				96.73 (95-98)	
**Type of experimental care**											.83		
	Fully automated	4		7		830		0.60 (−0.03 to 1.24)				57.23 (1-82)	
	Guided	14		15		4599		0.53 (0.25-0.81)^e^				93.19 (90-95)	

^a^Total sample analyzed: total randomized N in intent-to-treat analyses and N of completers in completers-only analyses.

^b^Two-tailed *P* value reflecting whether the difference in effect sizes between subgroups is significant.

^c^CPAP: continuous positive airway pressure.

^d^CPAP adherence operationalized as average nightly CPAP use in hours.

^e^*P* value is significant at the .05 level.

^f^Not applicable.

### Subgroup Analysis of eHealth Interventions and Continuous Positive Airway Pressure Adherence

The results of the subgroup analyses are shown in Table 1. No significant differences in CPAP adherence were found between studies investigating eHealth as an add-on to CAU (n=13) and studies investigating eHealth as a replacement of CAU (n*=*5) (see Multimedia Appendix 2 for an overview of both types of studies). A second subgroup analysis compared interventions providing eHealth only (n=11) [17,24,25,31,34,36-41] with blended approaches combining eHealth and face-to-face strategies (n=5) [26-30]. Two studies [32,33] were excluded, because it was unclear whether collaborative management was provided using eHealth technology. No significant differences between the subgroups were found (Table 1). A third analysis comparing the effectiveness of fully automated eHealth interventions (n=4) [24,25,37,39] versus guided eHealth interventions (n=14) [17,26-34,36,38,40,41] also found no significant differences (Table 1).

### Review of the Long-Term Follow-Up Effects of eHealth Interventions and Continuous Positive Airway Pressure Adherence

Four studies included follow-up assessments subsequent to the postintervention measurement. In view of this limited number of studies and their large variation in follow-up periods, no meta-analysis was conducted. We will now review the follow-up data, distinguishing between short-term follow-up (1-6 months; three studies) and long-term follow-up (≥1 year; two studies).

Regarding studies with short-term follow-up, Lo Bue et al [35] did not report 3- and 6-month follow-up data in detail. Lai et al [30] found that participants who received a brief motivational enhancement education program on top of CAU showed greater adherence at a 3-month follow-up (see Multimedia Appendix 2 for more details). Stepnowsky et al [33] found that average nightly CPAP use in hours was higher at a 4-month follow-up for participants who received a telemonitoring intervention with a Web-based portal for education and self-monitoring than for participants who received CAU consisting of preset contact with clinical staff (*P*=.03).

Regarding long-term follow-up, Bouloukaki et al [26] reported that telephone support supplemented to CAU was superior to CAU at a 2-year follow-up in terms of the range of CPAP adherence measures (see Multimedia Appendix 2 for more details). However, Lo Bue et al [35] found at a 1-year follow-up that telephone support adjunctive to CAU was not more effective than CAU in terms of increasing nightly CPAP use in hours.

## Discussion

### Principal Findings

To our knowledge, this meta-analytic review is the first to systematically assess the influence of eHealth interventions in improving adherence to CPAP treatment among adults with OSA. Nineteen eligible studies were identified, and our meta-analysis included data from 18 studies reporting 22 comparisons. A heterogeneous collection of eHealth interventions, employed either as add-ons or as replacements to CAU, were found to increase the average CPAP adherence by about half an hour a night as compared with CAU alone. No significant differences in effects emerged between eHealth provision supplemented to CAU and eHealth as a replacement of CAU. Additionally, no significant differences were found between other subgroups of approaches (eHealth only versus blended interventions and fully automated versus guided eHealth interventions).

In line with preliminary investigations [8,15], the results of the meta-analysis suggested the potential of a broad range of eHealth technologies as tools to promote and reinforce adherence to CPAP treatment for adults with OSA. eHealth technologies can help to deliver standardized education to patients and to closely monitor their daily-life CPAP data, enabling early detection of problems and nonadherence, followed by timely and appropriate response at a distance. This could have important clinical implications, potentially reducing the number of necessary follow-up visits to clinics and enhancing the numerous health benefits associated with CPAP treatment, such as improved sleep quality, improved sleep efficiency [5,42], and reduced blood pressure [43,44]. Many studies have furthermore identified dose-response relationships in the treatment of OSA with CPAP [8,45,46], demonstrating more hours of CPAP use to be associated with better outcomes. More specifically, patients with higher treatment adherence generally showed larger decreases in self-reported sleepiness, as well as greater improvements in functional outcomes owing to a reduced impact of excessive sleepiness on everyday activities. Overall, our meta-analysis showed that eHealth interventions are able to increase adherence to CPAP treatment, which can positively impact a range of health outcomes.

It is difficult to determine the clinical relevance of our meta-analytic finding that eHealth technologies increased average CPAP adherence by half an hour a night. There is no established general cut-off point defining how much adherence leads to clinically meaningful improvement. In contrast to the dose-response relationships noted above, some studies have reported effective treatment of OSA with relatively few hours of CPAP use, whereas others noted little progress at longer durations. Individual variation in CPAP response in terms of indicators, such as sleepiness, may depend on factors such as biological response mechanisms [46]. In other words, different individuals may experience different changes in their clinical symptoms in relation to their levels of CPAP adherence and relative improvement.

As to whether specific characteristics of eHealth adherence interventions could potentially moderate CPAP response, our meta-analysis showed no significant differences in effect sizes for eHealth adherence interventions delivered as (1) replacements to CAU rather than as add-ons, (2) blended versus eHealth-only strategies, or (3) guided versus fully automated interventions**.** These findings should be interpreted with care, as analyses may have been underpowered and varying types and intensities of CAU may have influenced the results independent of the eHealth interventions themselves. Future studies should therefore compare different eHealth adherence interventions directly within studies to shed light on the most effective types or components of such interventions. For example, a recent study conducted by Hwang et al [24] has assessed the individual effects of two types of eHealth interventions on adherence to CPAP treatment, as well as their combined effect. Adding a Web-based education program to CAU was not found to be effective in increasing adherence rates, whereas adding CPAP telemonitoring with automated patient usage feedback, as well as a combination of telemonitoring and Web-based education was found to successfully increase adherence. Direct comparisons of different eHealth strategies are also of interest because these differ widely in terms of implementation effort, complexity, and cost. Partially or fully automated eHealth components, for instance, would require no or substantially less involvement of clinical staff, with favorable clinical implications in terms of intervention cost and availability, as well as the allocation of health care resources.

### Study Limitations

Follow-up data beyond posttreatment measurements were too limited for meta-analytical assessment. Furthermore, the results are limited to adult populations scoring generally well above the threshold for severe OSA; it is unclear whether the results could be generalized to younger populations or those with less severe OSA. Another limitation was the moderate-to-high heterogeneity in the results between the included studies, as well as the high risk of bias in some studies for one or more dimensions. The type and intensity of CAU provided in the control condition varied considerably, potentially biasing the results. The null findings in our subgroup or moderator analyses should be interpreted with caution, as the analyses may have been underpowered. Further limitations lie in the fact that not all studies performed conventional polysomnography to diagnose patients and that CPAP may not have always been manually titrated. We did not search for gray literature, and we searched only for literature published after 2000. Finally, in several studies, routine or as-needed telephone support was part of CAU, whereas in other studies, it was confined to the experimental intervention condition.

### Directions for Future Research

Economic evaluations are needed to determine the cost-effectiveness of eHealth adherence interventions in comparison with CAU. To our knowledge, only two studies [34,41] have carried out such economic evaluations. The results of both these studies suggested the use of eHealth adherence interventions to produce effects similar to those of traditional care, with significant cost saving by, for example, reducing travel costs and productivity losses [34], and reductions in face-to-face visits to the sleep clinic [41]. Future studies could specifically adopt both societal and health care perspectives in examining cost-effectiveness in comparison with CAU.

Another future research direction would be to investigate the long-term effectiveness of eHealth interventions in improving adherence to CPAP treatment. What happens when patients are no longer monitored or followed up by visits to the clinic after their first months using the CPAP device?

Currently, little is known about which eHealth strategies or components are most effective in increasing CPAP adherence. Such information could help design the most efficient and effective interventions. Future studies could also investigate the benefits of eHealth adherence interventions for individuals with moderate levels of OSA.

With regard to methodology, future studies should carefully take into account the various risks of bias identified in many studies in this review, that is, outcome measures should be defined a priori and should be adequately reported, an intent-to-treat design should be adopted when analyzing the data, and any baseline imbalances should be adequately accounted.

Finally, an interesting direction for future research would be to examine the potential of incorporating psychological theories and models into eHealth adherence interventions. Promising results have already been reported for interventions based on cognitive-behavioral treatment principles [47] and motivational interviewing [48,49]. Such interventions can maximize adherence by focusing on negative or distorted beliefs or attitudes, outcome expectations, perceived self-efficacy, and motivational issues.

### Practical Implications

The current findings suggest that a broad range of eHealth interventions are effective in increasing adherence to CPAP treatment. Given the literature showing that higher CPAP adherence is generally associated with better outcomes, the potential of eHealth should be further explored and exploited. We therefore recommend assessing personal pathways in more detail to determine who can benefit the most from digitally enabled adherence support. Research is also needed on the cost-effectiveness of interventions and on how they might be implemented on a large scale.

### Conclusions

Providing eHealth interventions to adults with OSA during CPAP treatment can improve treatment adherence in the initial months, increasing the mean nightly duration of use by about half an hour. eHealth technologies can also be employed as tools to deliver standardized education and to monitor patients more closely in daily life. This enables the early detection of problems and nonadherence and allows timely and appropriate responses at a distance. More information is still needed about the specific types of eHealth interventions and the timing, duration, and intensity of eHealth interventions that health care providers could effectively implement.

## Appendix

Multimedia Appendix 1 Search string.

Multimedia Appendix 2 An overview of the relevant characteristics of each of the included studies (n=19).

## Abbreviations

APAP: automatically adjusted positive airway pressure
CAU: care as usual
COPD: chronic obstructive pulmonary disease
CPAP: continuous positive airway pressure
eHealth: electronic health
OSA: obstructive sleep apnea

## References

[1] American Academy of Sleep Medicine Task Force (1999). Sleep–Related Breathing Disorders in Adults: Recommendations for Syndrome Definition and Measurement Techniques in Clinical Research. Sleep.

[2] The Dutch College of General Practitioners (2014). Dutch College of General Practitioners’ guideline sleep problems and sleeping pills (second revision) [NHG-Standaard Slaapproblemen en slaapmiddelen (tweede herziening)]. Huisarts en wetenschap.

[3] (2005). International classification of sleep disorders: Diagnostic and coding manual (2nd ed).

[4] Senaratna CV, Perret JL, Lodge CJ, Lowe AJ, Campbell BE, Matheson MC, Hamilton GS, Dharmage SC (2017). Prevalence of obstructive sleep apnea in the general population: A systematic review. Sleep Med Rev.

[5] Giles T, Lasserson TJ, Smith BH, White J, Wright J, Cates CJ (2006). Continuous positive airways pressure for obstructive sleep apnoea in adults. Cochrane Database Syst Rev.

[6] Jonas DE, Amick HR, Feltner C, Weber RP, Arvanitis M, Stine A, Lux L, Harris RP (2017). Screening for Obstructive Sleep Apnea in Adults: Evidence Report and Systematic Review for the US Preventive Services Task Force. JAMA.

[7] Weaver TE, Sawyer AM (2010). Adherence to continuous positive airway pressure treatment for obstructive sleep apnoea: implications for future interventions. Indian J Med Res.

[8] Sawyer AM, Gooneratne NS, Marcus CL, Ofer D, Richards KC, Weaver TE (2011). A systematic review of CPAP adherence across age groups: clinical and empiric insights for developing CPAP adherence interventions. Sleep Med Rev.

[9] Weaver TE, Grunstein RR (2008). Adherence to Continuous Positive Airway Pressure Therapy: The Challenge to Effective Treatment. Proceedings of the American Thoracic Society.

[10] Hiensch R, Nandedkar DS, Feinsilver SH (2016). Optimizing CPAP Treatment for Obstructive Sleep Apnea. Curr Sleep Medicine Rep.

[11] Crawford MR, Espie CA, Bartlett DJ, Grunstein RR (2014). Integrating psychology and medicine in CPAP adherence--new concepts?. Sleep Med Rev.

[12] Mehrtash M, Bakker JP, Ayas N (2019). Predictors of Continuous Positive Airway Pressure Adherence in Patients with Obstructive Sleep Apnea. Lung.

[13] Wozniak D, Lasserson TJ, Smith I (2014). Educational, supportive and behavioural interventions to improve usage of continuous positive airway pressure machines in adults with obstructive sleep apnoea. Cochrane Database Syst Rev.

[14] Hwang D, Doctorian T (2017). Monitoring Progress and Adherence with PAP Therapy for OSA: the Roles of Telemedicine and Mobile Health Applications. Curr Pulmonol Rep.

[15] Pépin JL, Tamisier R, Hwang D, Mereddy S, Parthasarathy S (2017). Does remote monitoring change OSA management and CPAP adherence?. Respirology.

[16] Kwiatkowska M, Ayas N (2010). Can telemedicine improve CPAP adherence?. Thorax.

[17] Taylor Y, Eliasson A, Andrada T, Kristo D, Howard R (2006). The role of telemedicine in CPAP compliance for patients with obstructive sleep apnea syndrome. Sleep Breath.

[18] Covidence systematic review software.

[19] (2009). Introduction To Meta-analysis.

[20] Orsini N, Bottai M, Higgins J, Buchan I (2006). EconPapers.

[21] Duval S, Tweedie R (2000). Trim and fill: A simple funnel-plot-based method of testing and adjusting for publication bias in meta-analysis. Biometrics.

[22] Egger M, Davey Smith G, Schneider M, Minder C (1997). Bias in meta-analysis detected by a simple, graphical test. BMJ.

[23] Higgins JP, Green S (2011). Cochrane Handbook for Systematic Reviews of Interventions (Version 5.1.0).

[24] Hwang D, Chang JW, Benjafield AV, Crocker ME, Kelly C, Becker KA, Kim JB, Woodrum RR, Liang J, Derose SF (2018). Effect of Telemedicine Education and Telemonitoring on Continuous Positive Airway Pressure Adherence. The Tele-OSA Randomized Trial. Am J Respir Crit Care Med.

[25] Mendelson M, Vivodtzev I, Tamisier R, Laplaud D, Dias-Domingos S, Baguet JP, Moreau L, Koltes C, Chavez L, De Lamberterie G, Herengt F, Levy P, Flore P, Pépin JL (2014). CPAP treatment supported by telemedicine does not improve blood pressure in high cardiovascular risk OSA patients: a randomized, controlled trial. Sleep.

[26] Bouloukaki I, Giannadaki K, Mermigkis C, Tzanakis N, Mauroudi E, Moniaki V, Michelakis S, Siafakas NM, Schiza SE (2014). Intensive versus standard follow-up to improve continuous positive airway pressure compliance. Eur Respir J.

[27] Hui DS, Chan JK, Choy DK, Ko FW, Li TS, Leung RC, Lai CK (2000). Effects of augmented continuous positive airway pressure education and support on compliance and outcome in a Chinese population. Chest.

[28] Nilius G, Cottin U, Domanski U, van’t Hoog T, Franke K, Burian S, Ruehle K (2012). Effects of intensive outpatient training on the adherence of CPAP therapy for patients with OSA. Somnologie.

[29] Pengo MF, Czaban M, Berry MP, Nirmalan P, Brown R, Birdseye A, Woroszyl A, Chapman J, Kent BD, Hart N, Rossi GP, Steier J (2018). The effect of positive and negative message framing on short term continuous positive airway pressure compliance in patients with obstructive sleep apnea. J Thorac Dis.

[30] Lai AY, Fong DY, Lam JC, Weaver TE, Ip MS (2014). The efficacy of a brief motivational enhancement education program on CPAP adherence in OSA: a randomized controlled trial. Chest.

[31] Fields BG, Behari PP, McCloskey S, True G, Richardson D, Thomasson A, Korom-Djakovic D, Davies K, Kuna ST (2016). Remote Ambulatory Management of Veterans with Obstructive Sleep Apnea. Sleep.

[32] Stepnowsky CJ, Palau JJ, Marler MR, Gifford AL (2007). Pilot randomized trial of the effect of wireless telemonitoring on compliance and treatment efficacy in obstructive sleep apnea. J Med Internet Res.

[33] Stepnowsky C, Edwards C, Zamora T, Barker R, Agha Z (2013). Patient perspective on use of an interactive website for sleep apnea. Int J Telemed Appl.

[34] Isetta V, Negrín MA, Monasterio C, Masa JF, Feu N, Álvarez A, Campos-Rodriguez F, Ruiz C, Abad J, Vázquez-Polo FJ, Farré R, Galdeano M, Lloberes P, Embid C, de la Peña M, Puertas J, Dalmases M, Salord N, Corral J, Jurado B, León C, Egea C, Muñoz A, Parra O, Cambrodi R, Martel-Escobar M, Arqué M, Montserrat JM, SPANISH SLEEP NETWORK (2015). A Bayesian cost-effectiveness analysis of a telemedicine-based strategy for the management of sleep apnoea: a multicentre randomised controlled trial. Thorax.

[35] Lo Bue A, Salvaggio A, Isidoro SI, Romano S, Marrone O, Insalaco G (2014). Usefulness of reinforcing interventions on continuous positive airway pressure compliance. BMC Pulm Med.

[36] Sedkaoui K, Leseux L, Pontier S, Rossin N, Leophonte P, Fraysse J, Didier A (2015). Efficiency of a phone coaching program on adherence to continuous positive airway pressure in sleep apnea hypopnea syndrome: a randomized trial. BMC Pulm Med.

[37] Kuna ST, Shuttleworth D, Chi L, Schutte-Rodin S, Friedman E, Guo H, Dhand S, Yang L, Zhu J, Bellamy SL, Volpp KG, Asch DA (2015). Web-Based Access to Positive Airway Pressure Usage with or without an Initial Financial Incentive Improves Treatment Use in Patients with Obstructive Sleep Apnea. Sleep.

[38] Hoet F, Libert W, Sanida C, Van den Broecke S, Bruyneel A, Bruyneel M (2017). Telemonitoring in continuous positive airway pressure-treated patients improves delay to first intervention and early compliance: a randomized trial. Sleep Med.

[39] DeMolles DA, Sparrow D, Gottlieb DJ, Friedman R (2004). A pilot trial of a telecommunications system in sleep apnea management. Med Care.

[40] Fox N, Hirsch-Allen AJ, Goodfellow E, Wenner J, Fleetham J, Ryan CF, Kwiatkowska M, Ayas NT (2012). The impact of a telemedicine monitoring system on positive airway pressure adherence in patients with obstructive sleep apnea: a randomized controlled trial. Sleep.

[41] Turino C, de Batlle J, Woehrle H, Mayoral A, Castro-Grattoni A, Gómez S, Dalmases M, Sánchez-de-la-Torre M, Barbé F (2017). Management of continuous positive airway pressure treatment compliance using telemonitoring in obstructive sleep apnoea. Eur Respir J.

[42] Gay P, Weaver T, Loube D, Iber C, Positive Airway Pressure Task Force, Standards of Practice Committee, American Academy of Sleep Medicine (2006). Evaluation of positive airway pressure treatment for sleep related breathing disorders in adults. Sleep.

[43] Schein AS, Kerkhoff AC, Coronel CC, Plentz RD, Sbruzzi G (2014). Continuous positive airway pressure reduces blood pressure in patients with obstructive sleep apnea; a systematic review and meta-analysis with 1000 patients. Journal of Hypertension.

[44] Sun Y, Huang Z, Sun Q, Qiu L, Zhou T, Zhou G (2017). CPAP therapy reduces blood pressure for patients with obstructive sleep apnoea: an update meta-analysis of randomized clinical trials. Acta Cardiologica.

[45] Antic NA, Catcheside P, Buchan C, Hensley M, Naughton MT, Rowland S, Williamson B, Windler S, McEvoy RD (2011). The effect of CPAP in normalizing daytime sleepiness, quality of life, and neurocognitive function in patients with moderate to severe OSA. Sleep.

[46] Weaver TE, Maislin G, Dinges DF, Bloxham T, George CF, Greenberg H, Kader G, Mahowald M, Younger J, Pack AI (2007). Relationship between hours of CPAP use and achieving normal levels of sleepiness and daily functioning. Sleep.

[47] Richards D, Bartlett DJ, Wong K, Malouff J, Grunstein RR (2007). Increased adherence to CPAP with a group cognitive behavioral treatment intervention: a randomized trial. Sleep.

[48] Olsen S, Smith S, Oei T (2008). Adherence to continuous positive airway pressure therapy in obstructive sleep apnoea sufferers: a theoretical approach to treatment adherence and intervention. Clin Psychol Rev.

[49] Olsen S, Smith SS, Oei TP, Douglas J (2012). Motivational interviewing (MINT) improves continuous positive airway pressure (CPAP) acceptance and adherence: a randomized controlled trial. J Consult Clin Psychol.

