# Supplementary material for: Effectiveness of eHealth Interventions in Improving Treatment Adherence for Adults With Obstructive Sleep Apnea: Meta-Analytic Review
Source: J Med Internet Res. 2020 Feb 18;22(2):e16972. doi: 10.2196/16972 (PMC7055847; doi:10.2196/16972)
Supplement: Multimedia Appendix 1 [file jmir_v22i2e16972_app1.docx]

**Appendix 1: Search string**

***Search conducted on March 20^th^, 2018***

**PsycINFO (EBSCO)**

(DE "Compliance" OR DE "Treatment Compliance" OR DE “Treatment dropouts” OR TX(“fidelity” OR "complian*" OR "non-complian*" OR "noncomplian*" OR "adheren*" OR "non-adheren*" OR "nonadheren*" OR “dropout*” OR “drop-out*” OR “no-show*” OR “noshow*” OR “attend*” OR “non-attend*” OR “nonattend*” OR “absence*” OR “absent*” OR “non-appear*” OR “nonappear*”))AND (DE "Computer Assisted Therapy" OR DE "Telecommunications Media" OR DE "Electronic Communication" OR DE "Online Social Networks" OR DE "Online Therapy" OR DE "Social Media" OR DE "Telemedicine" OR DE "Text Messaging" OR DE “[Computer Mediated Communication](javascript:XslPostBack('ctl00$ctl00$MainContentArea$MainContentArea$xslResults','ThesaurusLink','LinkTarget%7CauthorityList%24LinkTerm%7CDE%2B%2522Computer%2BMediated%2BCommunication%2522');)” OR DE “Teleconferencing” OR DE “mobile devices” OR DE “communications media” OR DE “cellular phones” OR DE “Internet” OR DE “technology” OR DE “information technology” OR DE “virtual reality” OR DE “computer applications” OR TI(“Internet*” OR “Web*” OR “Online*” OR “tele*” OR “electronic*” OR “video*” OR “device*” OR “digital*” OR “software*” OR “mobile*” OR “technolog*” OR “e-health” OR “ehealth” OR “computer*” OR “e-treat*” OR “e-therap*” OR “mhealth” OR “m-health” OR “distance counsel*” OR “cybercounsel*” OR “cyber-counsel*” OR “cyber-treat*” OR “text-messag*” OR “textmessag*” OR “text messag*” OR “SMS*” OR “texting*” OR “short message service*” OR “smartphone*” OR “cell-phone*” OR “cellphone*” OR “cellular phone*” OR “blended*” OR “handheld device*” OR “hand held device*” OR “iPad*” OR “iPhone*” OR “email*” OR “e-mail*” OR “sensor*” OR “wearable*” OR “social media*” OR “social network*” OR “e-counsel*” OR “ecounsel*” OR “palmtop*” OR “telephone*” OR “WhatsApp” OR “Twitter” OR “Facebook” OR “Instagram” OR “forum” OR “chat*” OR “virtual reality*” OR “virtual-reality*” OR “avatar*” OR “Conversational agent*” OR “virtual coach” OR “virtual agent*” OR “embodied agent*” OR “avatar*” OR “relational agent*” OR “interactive agent*” OR “virtual character*” OR “virtual human*” OR “virtual assistant*”) OR AB(“Internet*” OR “Web*” OR “Online*” OR “tele*” OR “electronic*” OR “video*” OR “device*” OR “digital*” OR “software*” OR “mobile*” OR “technolog*” OR “e-health” OR “ehealth” OR “computer*” OR “e-treat*” OR “e-therap*” OR “mhealth” OR “m-health” OR “distance counsel*” OR “cybercounsel*” OR “cyber-counsel*” OR “cyber-treat*” OR “text-messag*” OR “textmessag*” OR “text messag*” OR “SMS*” OR “texting*” OR “short message service*” OR “smartphone*” OR “cell-phone*” OR “cellphone*” OR “cellular phone*” OR “blended*” OR “handheld device*” OR “hand held device*” OR “iPad*” OR “iPhone*” OR “email*” OR “e-mail*” OR “sensor*” OR “wearable*” OR “social media*” OR “social network*” OR “e-counsel*” OR “ecounsel*” OR “palmtop*” OR OR “telephone*” OR “WhatsApp” OR “Twitter” OR “Facebook” OR “Instagram” OR “forum” OR “chat*” OR “virtual reality*” OR “virtual-reality*” OR “avatar*” OR “Conversational agent*” OR “virtual coach” OR “virtual agent*” OR “embodied agent*” OR “avatar*” OR “relational agent*” OR “interactive agent*” OR “virtual character*” OR “virtual human*” OR “virtual assistant*”)) AND (DE “Asthma” OR DE “sleep apnea” OR DE “Chronic obstructive pulmonary disease” OR DE “Pulmonary Emphysema” OR TX(“Asthma*” OR “sleep apn*” OR “OSA*” OR “hypopnea*” OR “hypopnea*” OR “sleep disordered breath*” OR “COPD” OR “COAD” OR “chronic obstructive*” OR “chronic airflow obstruct*” OR “emphysema*” OR “chronic bronchitis” OR “chronic airway obstruct*” OR “obstructive pulmonary disease*” OR “obstructive respiratory disease*” OR “obstructive respiratory tract disease”))

Filters:

- Publication Year: 2000-2018
- Language: English or Dutch

**Pubmed**

("Treatment Adherence and Compliance"[Mesh:NoExp] OR "Patient Compliance"[Mesh] OR “Patient Dropouts”[Mesh] OR (fidelity[tiab] OR complian*[tiab] OR non-complian*[tiab] OR noncomplian*[tiab] OR adheren*[tiab] OR non-adheren*[tiab] OR nonadheren*[tiab] OR dropout*[tiab] OR drop-out*[tiab] OR no-show*[tiab] OR noshow*[tiab] OR attend*[tiab] OR non-attend*[tiab] OR nonattend*[tiab] OR absence*[tiab] OR absent*[tiab] OR non-appear*[tiab] OR nonappear*[tiab]))AND ( "Telemedicine"[Mesh] OR "Mobile Applications"[Mesh] OR "Social Media"[Mesh] OR "Therapy, Computer-Assisted"[Mesh:NoExp] OR "Drug Therapy, Computer-Assisted"[Mesh:NoExp] OR "Telecommunications"[Mesh:NoExp] OR "Electronic Mail"[Mesh] OR "Videoconferencing"[Mesh] OR "Cell Phone"[Mesh] OR "Distance Counseling"[Mesh] OR “Wearable Electronic Devices”[Mesh] OR “virtual reality”[Mesh] OR (internet*[tiab] OR web*[tiab] OR online*[tiab] OR computer*[tiab] OR electronic*[tiab] OR digital*[tiab] OR ehealth[tiab] OR e-health[tiab] OR e-treat*[tiab] OR e-therap*[tiab] OR mhealth[tiab] OR m-health[tiab] OR distance counsel*[tiab] OR cybercounsel*[tiab] OR cyber-counsel*[tiab] OR text-messag*[tiab] OR textmessag*[tiab] OR text messag*[tiab] OR SMS*[tiab] OR texting*[tiab] OR short message service*[tiab] OR mobile*[tiab] OR smartphone*[tiab] OR cell-phone*[tiab] OR cellphone*[tiab] OR cellular phone*[tiab] OR blended*[tiab] OR software app*[tiab] OR handheld device*[tiab] OR hand held device*[tiab] OR iPad*[tiab] OR iPhone*[tiab] OR email*[tiab] OR e-mail*[tiab] OR sensor*[tiab] OR wearable*[tiab] OR monitoring[tiab] OR social media*[tiab] OR social network*[tiab] OR e-counsel*[tiab] OR ecounsel*[tiab] OR palmtop*[tiab] OR telephone*[tiab] OR WhatsApp[tiab] OR Twitter[tiab] OR Facebook[tiab] OR Instagram[tiab] OR forum[tiab] OR chat*[tiab] OR virtual reality*[tiab] OR virtual-reality*[tiab] OR avatar*[tiab] OR Conversational agent*[tiab] OR virtual coach[tiab] OR virtual agent*[tiab] OR embodied agent*[tiab] OR avatar*[tiab] OR relational agent*[tiab] OR interactive agent*[tiab] OR virtual character*[tiab] OR virtual human*[tiab] OR virtual assistant*[tiab] OR tele-health [tiab] OR telehealth[tiab] OR tele-medicine[tiab] OR telemedicine[tiab] OR tele-care[tiab] OR telecare[tiab] OR tele-psychiatry[tiab] OR telepsychiatry[tiab] OR tele-guid*[tiab] OR teleguid*[tiab] OR tele-based[tiab] OR tele-deliver*[tiab] OR teledeliver*[tiab] OR tele-treat*[tiab] OR teletreat*[tiab] OR tele-therap*[tiab] OR telethera*[tiab] OR tele-intervention*[tiab] OR tele-counsel*[tiab] OR telecounsel*[tiab] OR tele-assist*[tiab] OR teleprevent*[tiab] OR tele-conferenc*[tiab] OR teleconferenc*[tiab] OR tele-monit*[tiab] OR telemonit*[tiab] OR tele-communicat*[tiab] OR telecommunicat*[tiab] OR tele-application*[tiab] OR tele-consult*[tiab] OR teleconsult*[tiab] OR video-guid*[tiab] OR videoguid*[tiab] OR video-mediated[tiab] OR video-based[tiab] OR videobased[tiab] OR video-deliver*[tiab] OR video-treat*[tiab] OR video-therap*[tiab] OR videothera*[tiab] OR video-intervention*[tiab] OR video-counsel*[tiab] OR video-assist*[tiab] OR video-conferenc*[tiab] OR videoconferenc*[tiab] OR video-monit*[tiab] OR videomonit*[tiab] OR video-communicat*[tiab] OR videocommunicat*[tiab] OR video-remind*[tiab] OR video-administered*[tiab] OR video-aided[tiab] OR video-application*[tiab] OR video-consult*[tiab] OR videoconsult*[tiab] OR video-enabled[tiab])) AND (“Asthma”[Mesh] OR “Sleep Apnea, Obstructive”[Mesh:NoExp] OR “Pulmonary Disease, Chronic Obstructive"[Mesh] OR “sleep apnea syndromes”[Mesh:NoExp] OR (Asthma*[tiab] OR sleep apn*[tiab] OR OSA*[tiab] OR hypopnea*[tiab] OR hypopnea*[tiab] OR sleep disordered breath*[tiab] OR sleep-disordered breath*[tiab] OR COPD[tiab] OR COAD[tiab] OR chronic obstructive*[tiab] OR chronic airflow obstruct*[tiab**]** OR emphysema*[tiab] OR chronic bronchitis[tiab] OR chronic airway obstruct*[tiab] OR obstructive pulmonary disease*[tiab] OR obstructive respiratory disease*[tiab] OR obstructive respiratory tract disease*[tiab]))

Filters:

- Publication Year: 2000-2018
- Language: English or Dutch
- Availability of full-text article
- Species: human

**Embase.com**

('patient compliance'/exp OR 'adherence'/exp OR 'dropouts'/exp OR 'patient dropout'/exp OR ‘patient attendance’/exp OR (‘fidelity’:ab,ti,kw OR ‘complian*’:ab,ti,kw OR ‘non-complian*’:ab,ti,kw OR ‘noncomplian*’:ab,ti,kw OR ‘adheren*’:ab,ti,kw OR ‘non-adheren*’:ab,ti,kw OR ‘nonadheren*’:ab,ti,kw OR ‘dropout*’:ab,ti,kw OR ‘drop-out*’:ab,ti,kw OR ‘no-show*’:ab,ti,kw OR ‘noshow*’:ab,ti,kw OR ‘attend*’:ab,ti,kw OR ‘non-attend*’:ab,ti,kw OR ‘nonattend*’:ab,ti,kw OR ‘absence*’:ab,ti,kw OR ‘absent*’:ab,ti,kw OR ‘non-appear*’:ab,ti,kw OR ‘nonappear*’:ab,ti,kw)) AND (‘telemedicine’/exp OR ‘telehealth’/exp OR ‘e-mail’/exp OR ‘mobile phone’/exp OR ‘social media’/exp OR ‘teleconference’/exp OR ‘text messaging’/exp OR ‘videoconferencing’/exp OR ‘mobile application’/exp OR ‘e-counseling’/exp OR ‘digital technology’/exp OR ‘mobile device’/exp OR ‘iphone’/exp OR ‘ipad’/exp OR 'computer assisted therapy'/de OR ‘monitoring’/exp OR ‘personal digital assistant’/exp OR ‘wearable sensor’/exp OR ‘wearable device’/exp OR ‘wearable technology’/exp OR ‘virtual reality’/exp OR ‘facebook’/exp OR ‘twitter’/exp **OR** (‘internet*’:ab,ti,kw OR ‘web*’:ab,ti,kw OR ‘online*’:ab,ti,kw OR ‘tele*’:ab,ti,kw OR ‘video*’:ab,ti,kw OR ‘computer*’:ab,ti,kw OR ‘electronic*’:ab,ti,kw OR ‘digital*’:ab,ti,kw OR ‘ehealth’:ab,ti,kw OR ‘e-health’:ab,ti,kw OR ‘e-treat*’:ab,ti,kw OR ‘e-therap*’:ab,ti,kw OR ‘mhealth’:ab,ti,kw OR ‘m-health’:ab,ti,kw OR ‘distance counsel*’:ab,ti,kw OR ‘cybercounsel*’:ab,ti,kw OR ‘cyber-counsel*’:ab,ti,kw OR ‘cyber-treat*’:ab,ti,kw OR ‘text-messag*’:ab,ti,kw OR ‘textmessag*’:ab,ti,kw OR ‘text messag*’:ab,ti,kw OR ‘SMS*’:ab,ti,kw OR ‘texting*’:ab,ti,kw OR ‘short message service*’:ab,ti,kw OR ‘mobile*’:ab,ti,kw OR ‘smartphone*’:ab,ti,kw OR ‘cell-phone*’:ab,ti,kw OR ‘cellphone*’:ab,ti,kw OR ‘cellular phone*’:ab,ti,kw OR ‘blended*’:ab,ti,kw OR ‘software app*’:ab,ti,kw OR ‘handheld device*’:ab,ti,kw OR ‘hand held device*’:ab,ti,kw OR ‘iPad*’:ab,ti,kw OR ‘iPhone*’:ab,ti,kw OR ‘email*’:ab,ti,kw OR ‘e-mail*’:ab,ti,kw OR ‘sensor*’:ab,ti,kw OR ‘wearable*’:ab,ti,kw OR ‘monitoring’:ab,ti,kw OR ‘social media*’:ab,ti,kw OR ‘social network*’:ab,ti,kw OR ‘e-counsel*’:ab,ti,kw OR ‘ecounsel*’:ab,ti,kw OR ‘palmtop*’:ab,ti,kw OR ‘telephone*’:ab,ti,kw OR ‘WhatsApp’:ab,ti,kw OR ‘Twitter’:ab,ti,kw OR ‘Facebook’:ab,ti,kw OR ‘Instagram’:ab,ti,kw OR ‘forum’:ab,ti,kw OR ‘chat*’:ab,ti,kw OR ‘virtual reality*’:ab,ti,kw OR ‘virtual-reality*’:ab,ti,kw OR ‘avatar*’:ab,ti,kw OR ‘Conversational agent*’:ab,ti,kw OR ‘virtual coach’:ab,ti,kw OR ‘virtual agent*’:ab,ti,kw OR ‘embodied agent*’:ab,ti,kw OR ‘avatar*’:ab,ti,kw OR ‘relational agent*’:ab,ti,kw OR ‘interactive agent*’:ab,ti,kw OR ‘virtual character*’:ab,ti,kw OR ‘virtual human*’:ab,ti,kw OR ‘virtual assistant*’:ab,ti,kw)) AND (‘Asthma’/exp OR ‘chronic bronchitis’/exp OR ‘chronic obstructive lung disease’/exp OR ‘sleep disordered breathing’/de OR ‘sleep apnea syndrome’/exp OR (‘Asthma*’:ab,ti,kw OR ‘sleep apn*’:ab,ti,kw OR ‘hypopnea*’:ab,ti,kw OR ‘hypopnoea*’:ab,ti,kw OR ‘sleep disordered breath*’:ab,ti,kw OR ‘sleep-disordered breath*’:ab,ti,kw OR ‘OSA*’:ab,ti,kw OR ‘COPD’:ab,ti,kw OR ‘COAD’:ab,ti,kw OR ‘chronic obstructive*’:ab,ti,kw OR ‘chronic airflow obstruct*’:ab,ti,kw OR ‘emphysema*’:ab,ti,kw OR ‘chronic bronchitis’:ab,ti,kw OR ‘chronic airway obstruct*’:ab,ti,kw OR ‘obstructive pulmonary disease*’:ab,ti,kw OR ‘obstructive respiratory disease*’:ab,ti,kw OR ‘obstructive respiratory tract disease*’:ab,ti,kw)) AND ([article]/lim OR [article in press]/lim OR [editorial]/lim OR [letter]/lim OR [review]/lim) AND ([dutch]/lim OR [english]/lim) AND [humans]/lim AND [embase]/lim AND [2000-2018]/py AND [embase]/lim NOT ([embase]/lim AND [medline]/lim)

Filters:

- Publication Year: 2000-2018
- EMBASE only
- Species: humans
- Language: English or Dutch
- Article type: article in press, article, editorial, review

**Cochrane library (Wiley)**

([mh “patient compliance”] OR [mh^“treatment adherence and compliance”] OR [mh “Patient dropouts”] OR (‘fidelity’:ab,ti,kw OR ‘complian*’:ab,ti,kw OR ‘non-complian*’:ab,ti,kw OR ‘noncomplian*’:ab,ti,kw OR ‘adheren*’:ab,ti,kw OR ‘non-adheren*’:ab,ti,kw OR ‘nonadheren*’:ab,ti,kw OR ‘dropout*’:ab,ti,kw OR ‘drop-out*’:ab,ti,kw OR ‘no-show*’:ab,ti,kw OR ‘noshow*’:ab,ti,kw OR ‘attend*’:ab,ti,kw OR ‘non-attend*’:ab,ti,kw OR ‘nonattend*’:ab,ti,kw OR ‘absence*’:ab,ti,kw OR ‘absent*’:ab,ti,kw OR ‘non-appear*’:ab,ti,kw OR ‘nonappear*’:ab,ti,kw)) AND ([mh “Telemedicine"] OR [mh "Mobile Applications"] OR [mh "Social Media"] OR [mh^"Therapy, Computer-Assisted"] OR [mh"Drug Therapy, Computer-Assisted"] OR [mh "Telecommunications"] OR [mh "Electronic Mail"] OR [mh "Videoconferencing"] OR [mh "Cell Phone"] OR [mh "Distance Counseling"] OR [mh “Wearable Electronic Devices”] OR [mh “virtual reality”] **OR** (‘internet*’:ab,ti,kw OR ‘web*’:ab,ti,kw OR ‘online*’:ab,ti,kw OR ‘tele*’:ab,ti,kw OR ‘video*’:ab,ti,kw OR ‘computer*’:ab,ti,kw OR ‘electronic*’:ab,ti,kw OR ‘digital*’:ab,ti,kw OR ‘ehealth’:ab,ti,kw OR ‘e-health’:ab,ti,kw OR ‘e-treat*’:ab,ti,kw OR ‘e-therap*’:ab,ti,kw OR ‘mhealth’:ab,ti,kw OR ‘m-health’:ab,ti,kw OR ‘distance counsel*’:ab,ti,kw OR ‘cybercounsel*’:ab,ti,kw OR ‘cyber-counsel*’:ab,ti,kw OR ‘cyber-treat*’:ab,ti,kw OR ‘text-messag*’:ab,ti,kw OR ‘textmessag*’:ab,ti,kw OR ‘text messag*’:ab,ti,kw OR ‘SMS*’:ab,ti,kw OR ‘texting*’:ab,ti,kw OR ‘short message service*’:ab,ti,kw OR ‘mobile*’:ab,ti,kw OR ‘smartphone*’:ab,ti,kw OR ‘cell-phone*’:ab,ti,kw OR ‘cellphone*’:ab,ti,kw OR ‘cellular phone*’:ab,ti,kw OR ‘blended*’:ab,ti,kw OR ‘software app*’:ab,ti,kw OR ‘handheld device*’:ab,ti,kw OR ‘hand held device*’:ab,ti,kw OR ‘iPad*’:ab,ti,kw OR ‘iPhone*’:ab,ti,kw OR ‘email*’:ab,ti,kw OR ‘e-mail*’:ab,ti,kw OR ‘sensor*’:ab,ti,kw OR ‘wearable*’:ab,ti,kw OR ‘monitoring’:ab,ti,kw OR ‘social media*’:ab,ti,kw OR ‘social network*’:ab,ti,kw OR ‘e-counsel*’:ab,ti,kw OR ‘ecounsel*’:ab,ti,kw OR ‘palmtop*’:ab,ti,kw OR ‘telephone*’:ab,ti,kw OR ‘WhatsApp’:ab,ti,kw OR ‘Twitter’:ab,ti,kw OR ‘Facebook’:ab,ti,kw OR ‘Instagram’:ab,ti,kw OR ‘forum’:ab,ti,kw OR ‘chat*’:ab,ti,kw OR ‘virtual reality*’:ab,ti,kw OR ‘virtual-reality*’:ab,ti,kw OR ‘avatar*’:ab,ti,kw OR ‘Conversational agent*’:ab,ti,kw OR ‘virtual coach’:ab,ti,kw OR ‘virtual agent*’:ab,ti,kw OR ‘embodied agent*’:ab,ti,kw OR ‘avatar*’:ab,ti,kw OR ‘relational agent*’:ab,ti,kw OR ‘interactive agent*’:ab,ti,kw OR ‘virtual character*’:ab,ti,kw OR ‘virtual human*’:ab,ti,kw OR ‘virtual assistant*’:ab,ti,kw)) AND ([mh“Asthma”] OR [mh^“obstructive sleep apnea”] OR [mh“Chronic obstructive pulmonary disease”] OR [mh^“sleep apnea syndromes”] OR (‘Asthma*’:ab,ti,kw OR ‘sleep apn*’:ab,ti,kw OR ‘hypopnea*’:ab,ti,kw OR ‘hypopnoea*’:ab,ti,kw OR ‘sleep disordered breath*’:ab,ti,kw OR ‘sleep-disordered breath*’:ab,ti,kw OR ‘OSA*’:ab,ti,kw OR ‘COPD’:ab,ti,kw OR ‘COAD’:ab,ti,kw OR ‘chronic obstructive*’:ab,ti,kw OR ‘chronic airflow obstruct*****’:ab,ti,kw OR ‘emphysema*’:ab,ti,kw OR ‘chronic bronchitis’:ab,ti,kw OR ‘chronic airway obstruct*’:ab,ti,kw OR ‘obstructive pulmonary disease*’:ab,ti,kw OR ‘obstructive respiratory disease*’:ab,ti,kw OR ‘obstructive respiratory tract disease*’:ab,ti,kw))

Filters:

- Publication year 2000-2018
- Word variations have been searched
- Limited to trials only
