# Supplementary material for: Effectiveness of eHealth Interventions in Improving Treatment Adherence for Adults With Obstructive Sleep Apnea: Meta-Analytic Review
Source: J Med Internet Res. 2020 Feb 18;22(2):e16972. doi: 10.2196/16972 (PMC7055847; doi:10.2196/16972)
Supplement: Multimedia Appendix 2 [file jmir_v22i2e16972_app2.docx]

An overview of the relevant characteristics of each of the included studies (*n* = 19).

NB: Reference studies #1 to #14 are ‘add-on’^1^ studies, whereas reference studies #15 to #19 are ‘replacement studies’^1^.

| **Study reference #1** | |
| --- | --- |
| Authors | Bouloukaki et al. |
| Year of publication | 2014 |
| Country | Greece |
| Study design | |
| Study conditions (*N*) | - UC (1550)  - UC + Intensive follow-up care (1550) |
| Measurements | - Baseline  - 1 Month  - 2 Years |
| Study population | |
| Age (*M, SD*) | - 55.6 ± 10.2  - 55.1 ± 10.7 |
| Gender (% female) | - 23.0  - 26.9 |
| Target population and recruitment strategy | Adults with OSAHS starting CPAP treatment, recruited through sleep disorders center. |
| Eligibility criteria | *Inclusion criteria:*  - Newly diagnosed OSAHS by PSG according to standard criteria  - Moderate- (AHI events/h ≥ 15 but < 30) to severe (AHI events/h ≥ 30) OSAHS  - No history of previous CPAP therapy  - An above-elementary school education  *Exclusion criteria:*  - Refusal of CPAP therapy  - Central sleep apnea syndromes  - Obesity hypoventilation syndrome  - Restrictive pulmonary and restrictive chest wall diseases  - Severe congestive heart failure  - History of life-threatening arrhythmias  - Severe cardiomyopathy,  - Long-term oxygen therapy  - Family or personal history of mental illness or alcohol abuse  - Severe cognitive impairment  - Concurrent oncological diseases  - History of narcolepsy or restless legs syndrome |
| Diagnostic procedure / OSA definition | AHI ≥ 15 events/h determined by single-night full diagnostic PSG study according to standard techniques, with monitoring of electroencephalogram (EEG), electro-oculogram, electromyogram, flow (by OroNasal thermistor and nasal air pressure transducer), thoracic and abdominal respiratory effort (by respiratory induction plethysmography), oximetry, and body position. PSG recordings were manually interpreted over 30-s periods, by skilled staff, in accordance with the American Academy of Sleep Medicine (AASM) 2007 guidelines. Determination of sleep stages and arousals was performed, according to the AASM 2007 criteria, using EEG montages including frontal, central and occipital leads. The definition of apnea and hypopnea followed the AASM standard criteria. |
| Interventions | |
| E-health condition |  |
| Add-on or replacement^1^ | Add-on |
| Type technology | Telephone |
| Type intervention | Usual follow-up care (see 'control condition') plus:  - Additional educational visit with partner or family required to accompany the patient. Visit included 15-minute videotape educational session covering a series of topics about OSAHS and CPAP, followed by a 10–15 min lecture from the sleep clinic’s registered nurses to reinforce the key concepts of the education session and the benefits of adherence.  - Instructed to complete sleep diary during first month.  - Telephone calls (day 2 and day 7) by nurse to check any concerns, problems, and discuss adherence. Home visit if doubting the latter.  - Review clinic (day 15 and 30) by sleep specialist, emphasizing adherence and addressing any concerns |
| Duration &  frequency | 1 educational session (+-30 minutes), 1-month sleep diary, 2 telephone calls, 2 visits to clinic |
| Control condition | Usual follow-up care: review visits (15-30 minutes) to clinic after 1 month, at 3-monthly intervals during first year, and every 6 months thereafter. During these visits: clinical assessment, encouragement CPAP use, discussion health issues related to the condition. Compliance data from CPAP device was reviewed and discussed. Any concerns or questions were addressed by CPAP clinic nurse. In addition, a 24-h treatment consultation telephone line to the sleep nurses was open. |
| Outcome(s) | |
| Assessment adherence | 1) Data measured by real-time clock in CPAP device and uploaded to a computer using specialised software.  2) Self-reported CPAP as recorder in sleep diary during first month of trial |
| Operationalization adherence | 1.1) % nights CPAP used  1.2) nr nights CPAP used a week  1.3) average nightly CPAP use in hours on nights being used  1.4) % regular users, defined as using CPAP for an average of ≥ 4 hours a night on ≥70% of nights |
| Results | |
| Effects (*M, SD*) on adherence, incl. significance (*p*-values) | 2-Year results:  1.1) Sign. (p<.001): 75.1 ± 23.9 VS 88.1 ± 8.2  1.2) Sign. (p<.001): 5.2 ± 2.3. VS 6.2 ± 3.9  1.3) Sign. (p<.001): 5.2 ± 2.2 VS 6.9 ± 1.8  1.4) Sign. (p<.001): 79.8 VS 92.8  1-Month results*:*  1.3) 6.4 ±1.1 VS 7.6 ±1.2  *Note: no significance testing for 1-month results* |
| Other | |
| Source of funding and competing interest | - |
| Study limitations and other comments | - |

| **Study reference #2** | |
| --- | --- |
| Authors | DeMolles et al. |
| Year of publication | 2004 |
| Country | Massachusetts, United States |
| Study design | |
| Study conditions (*N*) | - UC (15)  - UC + Telephone-linked communication (15) |
| Measurements | - Baseline  - 2 Months |
| Study population | |
| Age (*M, SD*) | - 42.0 ± 13.0  - 49.8 ± 15.7 |
| Gender (% female) | n/a |
| Target population and recruitment strategy | Adults with OSAS starting CPAP treatment, recruited through collaborating home care company. |
| Eligibility criteria | *Inclusion criteria:*  - Aged ≥ 18 years  - English-speaking  - Have a physician diagnosis of OSAS  - Have PSG demonstrating AHI ≥ 15 events/h of sleep  *Exclusion criteria:*  - Reported prior CPAP use |
| Diagnostic procedure / OSA definition | PSG demonstrating AHI ≥ 15 events/h |
| Interventions | |
| E-health condition |  |
| Add-on or replacement^1^ | Add-on |
| Type technology | Computer-based telephone-linked communication (TLC) technology, i.e. interactive voice response system |
| Type intervention | Usual medical follow-up care (see 'control condition') plus: Automated TLC monitoring of patients' self-reported behavior, and providing education and reinforcement through a structured dialogue. The content of the TLC was based on patterns of CPAP adherence (i.e. low adherence defined as nonuse of the CPAP, use for fewer than 4 hours per night on nights using CPAP, or use fewer than 5 nights per week (or fewer than 2 nights in the case of the 3-day call) and side-effect profiles. Routine printed reports with information on the frequency and duration of CPAP use, side effects, and OSAS symptoms, were sent to the patients’ physicians biweekly or in case of low adherence or side-effects. |
| Duration &  frequency | 1 call after 3 days, where after weekly call for period of 2 months. Routine printed reports to physician biweekly or in case of non-adherence. |
| Control condition | Usual follow-up care: not further specified. |
| Outcome(s) | |
| Assessment adherence | Pressure-time meters installed in CPAP machines. |
| Operationalization adherence | Average nightly CPAP use in hours over 2-month period. |
| Results | |
| Effects (*M, SD*) on adherence, incl. significance (*p*-values) | Non-sign. (*p*=.08): 2.9 ± 2.4 VS 4.4 ± 3.0 |
| Other | |
| Source of funding and competing interest | Supported by the VA Health Services Research and Development Service. |
| Study limitations and other comments | - |

| **Study reference #3** | |
| --- | --- |
| Authors | Fox et al. |
| Year of publication | 2012 |
| Country | Canada |
| Study design | |
| Study conditions (*N*) | - UC (n=36)  - UC + Telemonitoring (n=39) |
| Measurements | - Baseline  - 3 Months |
| Study population | |
| Age (*M, SD*) | - 55.2 ± 11.5  - 52.0 ± 10.8 |
| Gender (% female) | - 22.2  - 18.0 |
| Target population and recruitment strategy | Adults with moderate-to-severe OSA starting CPAP treatment, recruited through university sleep disorders program. |
| Eligibility criteria | *Inclusion criteria:*  - Aged ≥ 19 years  - Moderate to severe OSA (AHI ≥ 15 events/hr as demonstrated by PSG)  - Prescribed CPAP by regular sleep physician  *Exclusion criteria:*  - Active cardiopulmonary or psychiatric disease  - Previously treated for OSA  - Not having a telephone line in their bedroom  - Not being able to return for follow-up visits. |
| Diagnostic procedure / OSA definition | AHI ≥ 15 events/hr using the Chicago scoring criteria for the determination of apneas and hypopneas, according to the American Academy of Sleep Medicine. Diagnoses were made by respirologists by an overnight PSG. |
| Interventions | |
| E-health condition |  |
| Add-on or replacement^1^ | Add-on |
| Type technology | Telemonitoring, web-based portal, telephone (as-needed). |
| Type intervention | Usual follow-up care (see 'control condition') plus: Automated weekly telemonitoring of CPAP adherence, applied CPAP pressure, mask leaks, and residual AHI events were transferred daily to a website. Research coordinator reviewed this data weekly and contacted the patient by telephone in case of: mask leak > 40 L/min for greater than 30% of the night, < 4 hr of use for two consecutive nights, machine measured AHI > 10 events/h, and 90th percentile of pressure > 16 cm H2O. Within these calls, coordinator would inquire symptoms, arrange talk or visit with PAP coordinator if necessary, and deliver interventions to improve compliance (e.g. a different mask, chin strap, modifications of pressure settings, modifications of humidifier settings, saline nasal sprays). |
| Duration &  frequency | Weekly telemonitoring with subsequent phone call as needed (see 'type intervention') over 3-month period. |
| Control condition | Usual follow-up care: phone call 2 days after starting CPAP treatment to ask about progress, adherence, and any problems with the machine encountered. Return visit after 4-6 weeks with PAP coordinator and doctor, and PAP data were downloaded from patients' machines (i.e. PAP adherence, applied PAP pressure, mask leak, and residual respiratory events). Any problems with treatment were addressed at this time. Return visit after 8  weeks for downloading PAP data only. Return visit after 3 months for downloading data and seeing sleep specialist. |
| Outcome(s) | |
| Assessment adherence | A modem attached to the CPAP device (REMstar® Pro nasal CPAP device (Phillips Respironics Inc., Murrysville, PA)) was programmed to send physiologic information directly to a web-based database across the telephone line each morning. |
| Operationalization adherence | 1) Mean % nights CPAP used  2) Mean nightly CPAP use in minutes  3) Mean nightly CPAP use in minutes on nights being used |
| Results | |
| Effects (*M, SD*) on adherence, incl. significance (*p*-values) | 1) Non-sign. (p=.19): 45.9 ± 38.0 VS 55.9 ± 40.0  2) Sign. (p=.006): 105 ± 118 VS 191 ± 147  3) Sign. (p<.0001): 207 ± 106 VS 321 ± 80 |
| Other | |
| Source of funding and competing interest | This study was partially supported by a research grant from Phillips Respironics Inc. |
| Study limitations and other comments | Adherence in UC was low (1.75 hr/night).  Low adherence in UC might be explained by low baseline Epworth Sleepiness Scale Scores (ESS), suggesting that our patients were not particularly sleepy. Indeed, adherence was much greater in both study arms when only patients with a baseline ESS of 11 or greater were included. |

| **Study reference #4** | |
| --- | --- |
| Authors | Hoet et al. |
| Year of publication | 2007 |
| Country | Belgium |
| Study design | |
| Study conditions (*N*) | - UC (n=23, analyses conducted on subsample n=20)  - UC + Telemonitoring (n=23, analyses conducted on subsample n=17) |
| Measurements | - Baseline  - 3 Months |
| Study population | |
| Age (*M, SD*) | - 54.0 ± 14.0  - 59.0 ± 13.0 |
| Gender (% female) | - 43.0  - 83.0 * |
| Target population and recruitment strategy | Adults with OSAS starting CPAP treatment, recruited through sleeping unit of a university hospital. |
| Eligibility criteria | *Inclusion criteria:*  - Aged ≥ 18 years  - Recently diagnosed with OSAS with an AHI ≥ 20 events/h  *Exclusion criteria:*  - Previous exposure to CPAP therapy  - Mixed or predominantly central sleep apnea  - A planned trip abroad for > 3 weeks during the first 3 months of follow-up  - Language barriers  - Cognitive or psychiatric disorders making it difficult to comprehend information regarding CPAP therapy and  provide informed consent  - Significant comorbidities such as severe COPD or hypoventilation  syndromes |
| Diagnostic procedure / OSA definition | AHI ≥ 20 events/h according to the American Academy of Sleep Medicine 2012 scoring rules, as determined by an overnight PSG. |
| Interventions | |
| E-health condition |  |
| Add-on or replacement^1^ | Add-on |
| Type technology | Telemonitoring, web-based portal, telephone (as-needed). |
| Type intervention | Usual follow-up care (see 'control condition') plus: Automated telemonitoring of CPAP usage data, masks leaks, CPAP pressure, and residual apnea-hypopnea index. Analysis of patient data through web portal, and call and set-up visit in case of air leaks, low apnea-hypopnea index, or low adherence to CPAP. |
| Duration &  frequency | Daily telemonitoring and 2-weekly analysis of patient usage data over 3- month period. |
| Control condition | Usual follow-up care: written CPAP treatment instructions and the ability to contact sleep unit as often as needed in order to resolve problems regarding CPAP use. Also, 1-month CPAP treatment group-educational session, and a 1.5- and 3-month visit to the pneumologist. |
| Outcome(s) | |
| Assessment adherence | Telemonitoring throughT4P (SRETT medical, France), transmission of CPAP data through General Packet Radio Service (GPRS) network. Data sent to secured server and analyzed onT4P Vision Web Portal. For UC participants, CPAP data was collected and analyzed by software Rescan from Resmed. |
| Operationalization adherence | 1) Mean nightly CPAP use in hours  2) Total number of hours CPAP use |
| Results | |
| Effects (*M, SD*) on adherence, incl. significance (*p*-values) | 1) Sign. (p=.02): 5.7 ± 1.6 VS 4.2 ± 1.9  2) Sign. (p=.03): 507 ± 205 VS 387 ± 185 |
| Other | |
| Source of funding and competing interest | This research did not receive any specific grant from funding agencies in the public, commercial, or not-for-profit sectors. |
| Study limitations and other comments | Limited sample size / power. |

| **Study reference #5** | |
| --- | --- |
| Authors | Hui et al. |
| Year of publication | 2000 |
| Country | China |
| Study design | |
| Study conditions (*N*) | - UC: Basic CPAP education and support (n=54, 3-month analyses conducted on subsample n=52)  - UC + Augmented CPAP education and support (n=54, 3-month analyses conducted on subsample n=45) |
| Measurements | - Baseline  - 1 Month  - 3 Months |
| Study population | |
| Age (*M, SD*) | 45 ± 11 |
| Gender (% female) | 10.2 |
| Target population and recruitment strategy | Adults with OSA starting CPAP treatment, recruited through a respiratory and sleep clinic in a university hospital. |
| Eligibility criteria | *Inclusion criteria:*  - Consecutive, symptomatic patients with newly diagnosed OSA commencing nasal CPAP treatment |
| Diagnostic procedure / OSA definition | AHI ≥ 10 events per hour of sleep as shown by overnight PSG, plus self-reported sleepiness. Sleep stages were scored according to standard criteria by Rechtschaffen and Kales. Apnea was defined as cessation of airflow for > 10 seconds, and hypopnea was defined as a reduction of airflow ≥ 50% for >10 seconds plus an oxygen desaturation of > 4% or an arousal. |
| Interventions | |
| E-health condition |  |
| Add-on or replacement^1^ | Add-on |
| Type technology | Telephone |
| Type intervention | Basic CPAP education support (see 'control ondition') plus: extra education by 15-minute videotape by physician followed by additional educational session by respiratory nurses. Also, telephone support by nurses (day 1, day 2, and at weeks 1, 2, 3, 4, 8, and 12) to help sort out any technical problem and encourage the use of CPAP. Finally, early face-to-face reviews by physician at weeks 1 and 2. |
| Duration &  frequency | Video and additional educational session each 15 minutes. |
| Control condition | Basic CPAP education and follow-up support: 10-min CPAP education program by a respiratory nurse, in which patients also received a brochure on OSA and CPAP treatment in Chinese. Follow-up by physicians and nurses at the CPAP clinic at 1 and 3 months to deal with any problem with the CPAP device or mask fit, and CPAP pressure was adjusted if necessary. |
| Outcome(s) | |
| Assessment adherence | Microprocessor with dual time meters recorded both CPAP machine run time and time spent at effective pressure (measured by a mask pressure transducer recorder). Adherence data were downloaded into a personal computer using the Respironics Encore software (Respironics). |
| Operationalization adherence | 1) Subjective/self-report: Average nightly CPAP use in hours  2) Objective (see column above): Average nightly CPAP use in hours spent at effective pressure  3) Objective (see column above): % of participants using their CPAP machine for more than 4 hours per night for  70% of the nights. |
| Results | |
| Effects (*M, SD*) on adherence, incl. significance (*p*-values) | 1) 1-Month: non-sign. (p=.5): 6.4 ± 0.2 VS 6.6 ± 0.2  3-Month: non-sign. (p=.6): 6.5 ± 0.2 VS 6.3 ± 0.2  2) 1-Month: non-sign. (p=.4): 5.3 ± 0.2 VS 5.5 ± 0.2  3-Month: non-sign. (p=.98): 5.3 ± 0.3 VS 5.3 ± 0.2  3) 1-Month: non-sign. (p=.15): 71 ± 4 VS 79 ± 4  3-Month: non-sign. (p=.6): 71 ± 4 VS 74 ± 4 |
| Other | |
| Source of funding and competing interest | - |
| Study limitations and other comments | Baseline differences sleepiness (Epworth Sleepiness Scale): higher in augmented support condition.  There was also a technical failure with the Aria/Encore software, resulting in missing CPAP compliance data for 2 patients in the control group and 9 in the augmented support group at 12 weeks. |

| **Study reference #6** | |
| --- | --- |
| Authors | Hwang et al. |
| Year of publication | 2018 |
| Country | California, United States |
| Study design | |
| Study conditions (*N*) | - UC (n=354, analyses conducted on subsample n=129)  - UC + Tel-ED (n=380, analyses conducted on subsample n=163): telemedicine web–based education  - UC + Tel-TM (n=375, analyses conducted on subsample n=125): CPAP telemonitoring with automated patient  usage feedback  - UC + Tele-both (n=346, analyses conducted on subsample n=138): web-based education plus telemonitoring  with automated patient feedback |
| Measurements | - Baseline  - 3 Months |
| Study population | |
| Age (*M, SD*) | 49.± 12.5 |
| Gender (% female) | 51.0 |
| Target population and recruitment strategy | Adults with OSA who were prescribed CPAP, recruited through sleep center that served large medical center. |
| Eligibility criteria | *Inclusion criteria:*  - Aged ≥ 18 years  - No previous sleep testing or trial of OSA therapy  - Eligible for home sleep apnea testing (HSAT)  *Exclusion criteria:*  - At risk of other sleep disorders (e.g., severe insomnia)  - Significant cardiopulmonary disease (e.g., heart failure, chronic respiratory failure)  - English not indicated as their preferred language |
| Diagnostic procedure / OSA definition | AHI ≥ 5 events per hour. Sleep medicine physician triaged appropriate patients to HSAT after review of the referral information and electronic health record chart. HSAT classes (up to 13 people) were led by a sleep trained respiratory therapist and sleep technologist and provided interactive OSA education and individualized HSAT setup. After a one-night test, each patient returned for an individual appointment with a respiratory therapist to review the results in terms of OSA diagnosis. |
| Interventions | |
| E-health condition |  |
| Add-on or replacement^1^ | Add-on |
| Type technology | - Tel-ED: Web-based education portal  - Tel-TM: Telemonitoring unit & interactive voice response/automated feedback messaging platform, delivering  feedback messages to the patients by means of phone call, text messaging, or email (patient choice)  - Tel-Both: Both Tel-ED and Tel-TM |
| Type intervention | - Tel-ED: usual care and follow-up care (see 'control condition') plus:  Two interactive telemedicine educational programs. The first program was e-mailed 2 weeks before the 1-hour  group education class, and was about pathophysiology of OSA (including animated videos depicting airway  narrowing), health-related risks and impact on daytime vigilance, introduction to CPAP therapy, and details of  the assessment process.  After confirming OSA diagnosis by home sleep apnea test, OSA, a link to the second education program was e-  mailed during their 1-week CPAP trial, focused on how to properly use CPAP, potential benefits on health and  daytime vigilance, methods of acclimating, and equipment care instructions.  - Tel-TM: usual care and follow-up care (see 'control condition') plus: automated daily telemonitoring and  platform triggering automated feedback via text messaging, phone calls, e-mail, or combination of these  (patient preference) when CPAP usage thresholds were met (see column 'duration and frequency'). Messages  were automatically sent and provided encouragement to improve use or positively reinforcing successful  adherence.  - Tel-Both: Both the Tel-ED and Tel-TM as described above. |
| Duration &  frequency | - Tel-ED: Two web-based education programs of 15 minutes each,  - Tel-TM: Daily telemonitoring and automated feedback over 3-month period. Usage thresholds for messages: 1)  no CPAP data for 3 consecutive days, 2) CPAP usage <4h for 3 consecutive nights, and 3) a 30-day period during  the first 3 months of therapy in which CPAP use was >4 h/night on >70% of days (i.e. Medicare definition of  adherence). |
| Control condition | Usual care and follow-up care: 1-hour small group education class about OSA, home sleep apnea test, and CPAP treatment. After a trial of CPAP treatment for 1 week, those willing to continue CPAP were prescribed therapy and scheduled for a 3-month follow-up appointment. |
| Outcome(s) | |
| Assessment adherence | Autotitrating device (AirSense 10; ResMed Corp) wirelessly transmitted CPAP data daily via a cellular signal into a cloud database (U-Sleep; ResMed Corp). |
| Operationalization adherence | Primary outcomes:  1) Number of nights CPAP use  2) Average nightly CPAP use in hours on all nights  Secondary outcomes:  3) % of nights using CPAP  4) % of nights using CPAP for > 4 hours a night  5) Average nightly CPAP use in hours on nights being used |
| Results | |
| Effects (*M, SD*) on adherence, incl. significance (*p*-values) | 2) Non-sign. (p1=.10), Sign. (p2=.0002), Sign. (p3=.0002): 3.8 ± 2.5 VS 4.0 ± 2.4 VS 4.4 ± 2.2 VS 4.8 ± 2.3  3) Non-sign. (p1=.28), Sign. (p2<.0001), Sign. (p3=.0004): 64.8 ± 34.2 VS 68.6 ± 31.3 VS 76.6 ± 28.3 VS 78.3 ± 28.3  5) Non-sign. (p1=.13), Sign. (p2=.006), Sign. (p3=.003): 5.2 ± 1.8 VS 5.2 ± 1.8 VS 5.3 ± 1.7 VS 5.8 ± 1.6  *Note 1: P-values compare estimated means from each intervention group (p1=Tele-ED, p2=Tele-TM, p3=Tele-both) to UC.*  *Note 2: Only between-group effects of experimental conditions versus UC are reported on, whereas not being compared to each other* |
| Other | |
| Source of funding and competing interest | - |
| Study limitations and other comments | Selective outcome reporting (see 'operationalization adherence' and 'results'). |

| **Study reference #7** | |
| --- | --- |
| Authors | Kuna et al. |
| Year of publication | 2015 |
| Country | Pennsylvania, United States |
| Study design | |
| Study conditions (*N*) | - UC (n=52)  - UC + Web-based access CPAP data (n=46)  - UC + Web-based access CPAP data + financial incentive (n=40) |
| Measurements | - Baseline  - 1 Week  - 3 Months |
| Study population | |
| Age (*M, SD*) | 50.9 ± 12.1 |
| Gender (% female) | 39.0 |
| Target population and recruitment strategy | Adults with OSA starting PAP treatment, recruited through sleep centers at 4 hospitals/medical centers. |
| Eligibility criteria | *Inclusion criteria:*  - Aged ≥ 18 years  - Prescribed PAP treatment for newly diagnosed OSA with an AHI of ≥ 10 events/h on in-laboratory PSG  - Access to a telephone and the Internet on all days of the week.  - Stable medical history in previous 2 months: no hospitalizations or new medical diagnoses other than OSA  - No change in medications and no regular use (> 3 times per week) of sedative or hypnotic medications in the  previous 2 months  *Exclusion criteria:*  - Diagnosis of another sleep disorder in addition to OSA  - Previous medical or surgical treatment for OSA  - Required supplemental oxygen or bilevel PAP treatment  - Worked rotating or night shift in the past 3 months  - Claustrophobia or facial pathology that prevented PAP treatment.  - Recent change in medication(s) |
| Diagnostic procedure / OSA definition | AHI ≥ 10 events/h on in-laboratory PSG. The PSGs were scored and interpreted by the sleep center staff at each clinical site using American Academy of Sleep Medicine recommended criteria. |
| Interventions | |
| E-health condition |  |
| Add-on or replacement^1^ | Add-on |
| Type technology | Web-based portal with CPAP data access |
| Type intervention | - Usual care (see 'control condition') plus Web-based access to CPAP data:  Ability to log into website at any time throughout the 3-month period to view a numerical and bar graph  display of daily hours of PAP use (i.e., mask-on time), both from the beginning of treatment and over the  previous 2 weeks of treatment. When logging in, patients were asked to indicate (yes/no) if they had used the  treatment for ≥4 hours in the past 24 h. This response was used to verify that they had logged into the website  on a particular day.  - Financial incentive:  Participants were informed that they could earn $30 for each day in the 1st week that they logged into the  website and had used PAP treatment for ≥4 hours in the previous night (max. first week $210). Payment  was made at the end of the first week of treatment. |
| Duration &  frequency | Web access to CPAP usage data over period of 3 months. |
| Control condition | Usual follow-up care at sleep centers: 1 clinic visit with the sleep specialist within 1 to 3 months of starting treatment. During this visit, specialist had access to PAP website for clinical management, and could share PAP information with the patient. The home health care company supplying the PAP equipment conducted routine follow-up care during the 3-month intervention, which generally consisted of a phone call to the patient at 1 week, 1 month, and 3 months. In case of mask or equipment problems, the home healthcare company provided routine clinical care. Finally, all participants completed questionnaires at baseline, and 1 and 3 months, for which they received $30 each. |
| Outcome(s) | |
| Assessment adherence | Adherence data was transmitted from APAP and CPAP devices (System One, Philips Respironics, Inc, Murrysville, PA) equipped with a wireless modem. On a daily basis, the modem transmitted data from the PAP unit to the manufacturer’s server, which in turn exported the results to the Way to Health website. |
| Operationalization adherence | 1) Mean nightly CPAP use in hours  2) Mean nights used  3) Mean nightly CPAP use in hours on nights being used |
| Results | |
| Effects (*M, SD*) on adherence, incl. significance (*p*-values) | 1-Week results:  1) Sign. (p1<.0001 & p2<.0001): 4.7 ± 3.3 VS 6.3 ± 2.5 VS 5.9 ± 2.5  2) Sign. (p1<.0001 & p2=.005): 5.5 ± 2.6 VS 6.7 ± 0.8 VS 6.6 ± 1.3  3) Sign. (p1=.002) & Non-Sign. (p2=.13): 6.0 ± 2.4 VS 6.6 ± 2.2 VS 6.3 ± 2.0  3-Month results:  1) Sign. (p1=<.0001 & p2=<.0001): 3.8 ± 3.3 VS 5.0 ± 3.2 VS 4.8 ± 3.0  2) Sign. (p1=<.0001 & p2=<.0001): 4.7 ± 3.0 VS 5.6 ± 2.3 VS 5.6 ± 2.3  3) Sign. (p1=<.0001 & p2=<.0001): 5.6 ± 2.4 VS 6.2 ± 2.3 VS 5.9 ± 2.0  *Note: P-values compare estimated means from each intervention group (p1=Web access and p2=Web access with financial incentive) to usual care* |
| Other | |
| Source of funding and competing interest | Funding: NIH RC2-AG036592-01; NIH 1P01-1HL094307. P’unk Ave, Inc., a Web-based software company in Philadelphia, PA, created the Way to Health website for the University of Pennsylvania and continued to support the website during the project. First and fourth author received grant support from Philips Respironics.  Last author was a Principal at VAL Health (Philadelphia, PA). Eleventh author has received research support from Humana, Weight Watchers, and CVS, and has consulted for Val Health. |
| Study limitations and other comments | About 80% of subjects in UC and UC + web-access to CPAP data groups were treated with APAP instead of CPAP, whereas APAP was used in 62% of participants in UC+ web-access to CPAP data + financial incentive group.  A greater % of participants with VS without the financial incentive viewed their PAP data in 1st week. This suggests participants in the financial incentive group to be aware of the requirements to qualify for reward. Possibly, the relatively higher adherence in participants with Web access but without financial incentive may have resulted in a ‘ceiling effect’ that prevented the financial incentive from having any additional benefit.  Finally, the two experimental interventions were not statistically compared. |

| **Study reference #8** | |
| --- | --- |
| Authors | Lai et al. |
| Year of publication | 2014 |
| Country | China |
| Study design | |
| Study conditions (*N*) | - UC (n=51)  - UC + brief motivational enhancement education program (n=49) |
| Measurements | - Baseline  - 1 Week  - 1 Month  - 3 Months |
| Study population | |
| Age (*M, SD*) | - 51 ± 10  - 53 ± 10 |
| Gender (% female) | - 82.3  - 83.7 |
| Target population and recruitment strategy | Adults with OSA starting CPAP treatment, recruited through sleep disorder center in hospital. |
| Eligibility criteria | *Inclusion criteria:*  - Aged ≥ 18 years  - Newly diagnosed OSA (AHI ≥ 5)  - Receiving in-laboratory auto-CPAP titration for the first time  - No prior OSA or CPAP education classes.  *Exclusion criteria:*  - Central sleep apnea  - Periodic leg movement disorders  - Coexisting COPD  - Pregnancy  - Psychiatric illness on treatment  - Cognitive impairment  - Illiteracy  - Unstable health conditions, e.g. end-stage renal failure on renal replacement therapy, malignancy currently on  radiotherapy or chemotherapy, or dependence in daily care  - Unable to attend the education session before discharge from sleep disorders center after CPAP titration  - Scheduled for OSA follow-up in other hospitals  - Participating in another clinical trial |
| Diagnostic procedure / OSA definition | AHI ≥ 5 as shown by overnight CPAP titration procedure.  *Note: randomization was stratified into three severity groups: AHI ≥ 5, AHI ≥ 15 and < 30, and AHI > 30* |
| Interventions | |
| E-health condition |  |
| Add-on or replacement^1^ | Add-on |
| Type technology | Telephone |
| Type intervention | Usual care (see 'control condition') plus brief motivational enhancement education program directed at enhancing that patients' perception of the risk of OSA, confidence in the ability to apply CPAP treatment (self-efficacy), and association of their behavior to the desired outcome (adherence) or outcome expectancy. Program included 25-min video and a booklet providing information and education including real-life experience of CPAP user. Also, 20-minute patient-centered interview was conducted based on motivational interviewing techniques: (1) using importance and confidence rulers to explore the barriers and facilitators of using CPAP (2) using a decision matrix to discuss the positive and negative aspects of using or not using CPAP (3) looking forward to the expected outcomes or benefits of using CPAP. Finally, 10-minute telephone follow-up to provide early review at day 2 of CPAP treatment (e.g. ask about experiences CPAP, discuss any problems encountered, highlight positive changes, encouraging adherence). |
| Duration &  frequency | Video and motivational interview (25 & 20 minutes respectively), plus 1 telephone call after 2 days |
| Control condition | Usual care comprised training/education session (30-minutes) and advice (15 minutes) on OSA and CPAP treatment. |
| Outcome(s) | |
| Assessment adherence | Adherence data was downloaded from CPAP devices with software. |
| Operationalization adherence | Primary outcome:  1) Mean nightly CPAP use in hours  Secondary outcomes:  2) % adherers, with adherent defined as using CPAP for ≥4 hours a night on at least 70% of nights  3) Intention to use: % nights on which CPAP has been switched on  4) Usage index: % days nights CPAP for at least 4 hours a night |
| Results | |
| Effects (*M, SD*) on adherence, incl. significance (*p*-values) | 1) Sign. (p<.001): 2.9 ± 2.5 VS 5.5 ± 1.8 (1 week), 2.6 ± 2.3 VS 4.8 ± 1.6 (1 month), 2.4 ± 2.3 VS 4.4 ± 1.8 (3  months)  2) Sign. (p<.001): 15 ± 29 VS 32 ± 63 (1 week), 10 ± 20 VS 30 ± 59 (1 month), 10 ± 20 VS 20 ± 41 (3 months)  3) Sign. (p<.001): 59 ± 36 VS 89 ± 19 (1 week), 52 ± 36 VS 84 ± 20 (1 month), 46 ± 36 VS 79 ± 23 (3 months)  4) Sign. (p<.001): 39 ± 38 VS 74 ± 29 (1 week), 34 ± 33 VS 70 ± 26 (1 month), 32 ± 32 VS 61 ± 28 (3 months) |
| Other | |
| Source of funding and competing interest | Conflicts of interest: Dr Lai has been sponsored to attend Sleep and Breathing Conference 2013 and 18th Congress of the Asian Pacific Society of Respirology by ResMed and Koninklijke Philips N.V ., respectively. Dr Lam has been sponsored to attend World Sleep Conference 2011, World Congress of Sleep Apnea 2012, and Sleep and Breathing Conference 2013 by ResMed, Koninklijke Philips N.V., and Homecare Medical Ltd, respectively. Dr Weaver is the member of the board of directors of ViMedicus, Inc. She has received research support from Teva Pharmaceuticals Industries, Ltd and has received equipment for her research from Koninklijke Philips N.V. She has been a consultant for Apnex Medical, Inc, and has received royalty fees for use of the Functional Outcomes of Sleep Questionnaire from NovaSom, Apnex Medical, Inc, GlaxoSmithKline, Koninklijke Philips N.V.,Cephalon, Inc (now Teva Pharmaceuticals Industries, Ltd), and Nova Nordisk. Dr Ip has received honoraria from Koninklijke Philips N.V. for a lecture in World Sleep 2011 and a lecture at Kyoto University in 2011. |
| Study limitations and other comments | Statistics on time contrasts not reported (only whole study period, hence no between group comparisons for 1 week, 1 month, and 3 months, only whole study period), and no other statistics than p-value and confidence intervals reported. Restricted eligibility criteria. |

| **Study reference #9** | |
| --- | --- |
| Authors | Lo Bue et al. |
| Year of publication | 2014 |
| Country | Italy |
| Study design | |
| Study conditions (*N*) | - UC (n=20, 12-month-analyses conducted on subsample n=18)  - UC + Extra early support (n=20, 12-month-analyses conducted on subsample n=19) |
| Measurements | - Baseline  - 3 Months  - 6 Months  - 12 Months |
| Study population | |
| Age (*M, SD*) | - 55.7 ± 8.3  - 58.6 ± 13.2 |
| Gender (% female) | 32.5 |
| Target population and recruitment strategy | Adults with OSA and indication to CPAP treatment, recruited through sleep disordered breathing center of IBIM-CNR Institute of Biomedicine and Molecular Immunology (IBIM), National Research Council. |
| Eligibility criteria | *Inclusion criteria:*  - Aged >18 years  - Diagnosis of OSA  - Indication for CPAP treatment according to international guidelines (incl. American Sleep Disorders Association)  *Exclusion criteria:*  - Impairments or comorbidities considered likely to interfere with adherence to instructions: neuromuscular  disease, unstable psychiatric disease or cognitive impairment, myocardial infarction, unstable angina, cardiac  failure, cerebrovascular accident, lung disease with awake resting oxygen saturation of less than 90%. |
| Diagnostic procedure / OSA definition | Apneas were identified on the airflow signal, and were classified as obstructive, central, or mixed, according to behavior of thoraco-abdominal movements. Hypopneas were scored when a ≥30% reduction in the airflow signal was detected in association with a reduction ≥4% of oxyhemoglobin saturation (SaO2). AHI was calculated as the number of (apneas + hypopneas)/h of recording that was analyzed. Time with SaO2 below 90% (TSat ≤90%) was calculated. |
| Interventions | |
| E-health condition |  |
| Add-on or replacement^1^ | Add-on |
| Type technology | Telephone |
| Type intervention | Usual follow-up care (see 'control condition') plus: Standardized daily telephone interview from sleep doctor in first week and after 1 month. During the interview patients were asked about the most common adverse events during CPAP treatment. Doctor reviewed progress and gave advice to manage CPAP-related adverse effects, and encouraged to maintain adherence to therapy. Besides, when necessary, technical support was given by the home care provider. |
| Duration &  frequency | Two standardized daily telephone interviews at day 1 and 30. |
| Control condition | Usual follow-up care: Patients were provided with a telephone number to call the doctor of the sleep center for support within office hours. The home care provider visited all patients at their home at month 3, 6, 12 from the start of therapy, and each time downloaded data from the device memory (time of device use per night), conducted other assessments, and transmitted all data to the sleep center. |
| Outcome(s) | |
| Assessment adherence | Adherence data was downloaded from CPAP machines (Weinmann SOMNOcomfort 2e) |
| Operationalization adherence | 1) Mean nightly CPAP use in hours  2) Monthly average number of nights of CPAP therapy ≥ 4 hours  3) % adherence (adherent defined as CPAP use ≥ 4 hours a night for at least 70% of the nights |
| Results | |
| Effects (*M, SD*) on adherence, incl. significance (*p*-values) | 1-Year results:  1) Non-sign. (p-value not reported): 3.8 VS 4.3  1-Month results:  2) Sign. (p=.02): 16.0 VS 23.2  3) Sign. (p-value not reported): 55.7 VS 77.5  *Note: 1-month differences in adherence became non-significant at the 2nd, 3rd month and at the 2nd (3) 58.3 VS 66.7), 3rd and 4th quarter (3) 56.3 VS 54.3) (statistics not provided).* |
| Other | |
| Source of funding and competing interest | Supported by the Italian National Research Council order numbers ME. P01.014.002 and ME.P01.014.009. |
| Study limitations and other comments | - |

| **Study reference #10** | |
| --- | --- |
| Authors | Mendelson et al. |
| Year of publication | 2014 |
| Country | France |
| Study design | |
| Study conditions (*N*) | - UC (n=53)  - UC + Telemedicine care (n=54) |
| Measurements | - Baseline  - 1 Month  - 4 Months |
| Study population | |
| Age (*M, SD*) | 63 ± 9 |
| Gender (% female) | 16.8 |
| Target population and recruitment strategy | Adults with OSA with high cardiovascular risk starting CPAP, recruited through referrals from multiple sleep centers by general practitioners and hospital specialists. |
| Eligibility criteria | *Inclusion criteria:*  - Aged 18-85 years old  - Diagnosed OSA (AHI > 15 events an hour)  - BMI < 40  - Cardiovascular risk SCORE > 5% or being in secondary prevention with a past history of cardiovascular disease  (transient ischemic attack, stroke, cerebral hemorrhage, myocardial infarction, angina, coronary  revascularization, arteriopathy, aortic aneurism)  *Exclusion criteria:*  - Central sleep apnea syndrome  - Cardiovascular score < 5%  - Cardiac failure  - History of hypercapnic chronic respiratory failure  - Incapacitated patients  - Pregnancy in accordance with article L 1121-6 of the French public health code  - Taking part in another clinical trial |
| Diagnostic procedure / OSA definition | AHI ≥ 15 events per hour of sleep. OSA diagnosis was obtained by full PSG or by simplified polygraph without electroencephalogram (EEG) recordings. Sleep was scored manually according to standard criteria. PSG used continuous acquisition of the following recordings: electroculogram (EOG; 3 channels), EEG (3 channels), electromyogram (1 channel) and electrocardiogram (1 channel). Airflow was measured using nasal pressure associated with the sum of oral and nasal thermistor signals. Respiratory effort was monitored with abdominal and thoracic bands. An apnea was defined as a complete cessation of airflow ≥ 10 s and a hypopnea as a reduction ≥ 50% in the nasal pressure signal or a decrease between 30% and 50% associated with either oxygen desaturation ≥ 4% or EEG arousal. Apneas were classified as obstructive, central, or mixed according to the presence or the absence of respiratory effort. |
| Interventions | |
| E-health condition |  |
| Add-on or replacement^1^ | Add-on |
| Type technology | Smartphone + application providing self-monitoring and self-care messages |
| Type intervention | Usual follow-up care (see 'control condition') plus: Patients received smartphone with an application designed to transmit clinical information. Patients transmitted self-measured morning and evening blood pressure (3-day measurements), CPAP adherence, and subjective sleepiness weekly through a questionnaire-based application. Quality of life questionnaires were transmitted monthly. Patients received daily pictograms with diet and physical-activity related messages on their smartphones. |
| Duration &  frequency | Daily self-monitoring and self-care messages for period of 4 months |
| Control condition | Usual follow-up care: Patients were contacted after 2 days to ask about adherence, side effects, and any problems encountered with the machine. After 4 weeks of treatment, patients met with their sleep specialist and adherence data was transferred from their machines. After 4 months, data were transferred again and patients saw their sleep specialist and were re-evaluated. |
| Outcome(s) | |
| Assessment adherence | Data was downloaded from CPAP machines |
| Operationalization adherence | Mean nightly CPAP use in minutes |
| Results | |
| Effects (*M, SD*) on adherence, incl. significance (*p*-values) | 4-Month results: Non-sign. (p-value not reported): 250 ± 166 VS 187 ± 178 |
| Other | |
| Source of funding and competing interest | This study was supported by a grant from Initiatives pour la Santé Domicile. Funders of the trial had no role in study design, data collection, data analysis or writing of the report. |
| Study limitations and other comments | Adherence was secondary outcome measure and study was not adequately powered for secondary outcome measures.  it is possible that telemedicine was perceived as an additional burden associated with the self-management of blood pressure and CPAP by patients randomized to this group. In fact, there were more dropouts in the telemedicine group than standard care (n = 8, 14.8% vs n = 1, 1.9%, respectively) |

| **Study reference #11** | |
| --- | --- |
| Authors | Nilius et al. |
| Year of publication | 2012 |
| Country | Germany |
| Study design | |
| Study conditions (*N*) | - UC (n=42, analyses conducted on subsample n=36)  - UC + Intensive education (n=42, analyses conducted on subsample n=40) |
| Measurements | - Baseline  - 3 Months |
| Study population | |
| Age (*M, SD*) | - 55.1 ± 12.0  - 49.8 ± 12.1 |
| Gender (% female) | - 36.1  - 40.0 |
| Target population and recruitment strategy | Adults with OSAS starting CPAP treatment, recruited through sleep laboratory program as part of a multi-level diagnostic investigation (medical history of increased daytime sleepiness and an RDI > 5 on a non-attended polygraph test). |
| Eligibility criteria | *Inclusion criteria:*  - Adults with OSAS undergoing CPAP for the first time  *Exclusion criteria:*  - AHI > 20% of the events  - Previous operations of pharyngeal structures  - Apparent heart failure and malignant cardiac arrhythmia  - Insufficient knowledge of the German language |
| Diagnostic procedure / OSA definition | Overnight attended PSG, which was evaluated by an experienced physician. The sleep stages and arousals were categorized in accordance with criteria of Rechtschaffen and Kales and recommendations of the American Sleep Disorders Association. Arousals were classified as respiratory if they occurred at the beginning of, or within 2 s, of apnea or hypopnea. If the flow signal was reduced by more than 50 % vis-à-vis in comparison with the initial signal, for more than 10 s, the episode was classified as hypopnea; if the amplitude of the flow signal was less than 20 % of the initial value, the episode was classified as apnea. |
| Interventions | |
| E-health condition |  |
| Add-on or replacement^1^ | Add-on |
| Type technology | Telephone |
| Type intervention | Usual education/follow-up care (see 'control condition') plus: Phone call by a specially trained non-medical employee once a week, with a planned total of 12 phone contacts. Any problems that had arisen were discussed. After 6 weeks, invitation to attend follow-up appointment at the clinic, which included a group briefing by a doctor, an individual consultation with a doctor, and a troubleshooting session regarding masks and devices by a trained technician. The data saved on the devices were read and the symptoms experienced during the day were assessed in accordance with the Epworth Sleepiness Scale. |
| Duration &  frequency | Weekly telephone consultation over period of 3 months, 6-week clinic visit (i.e. training session) |
| Control condition | Usual education/follow-up care included instruction to consult GP or sleep laboratory in case of problems. After 12 weeks, an overall summary of CPAP use was made. |
| Outcome(s) | |
| Assessment adherence | Reading off data from CPAP device. Recording platform Alice (Respironics). |
| Operationalization adherence | 1) Mean nightly CPAP use in hours  2) Mean nightly CPAP use in hours when considering only the patients using CPAP for >1 hour per night |
| Results | |
| Effects (*M, SD*) on adherence, incl. significance (*p*-values) | 1) Non-sign. (p-value not reported): 3.8 ± 3.0 VS 3.7 ± 2.2  2) Non-sign. (p-value not reported): 4.6 ± 2.7 VS 4.3 ± 1.8 |
| Other | |
| Source of funding and competing interest | - |
| Study limitations and other comments | Authors mention strict exclusion criteria as limitation: maybe a more problematic group of patients, e.g. those with cardiovascular diseases, would have the highest benefit of a more intensive training program. |

| **Study reference #12** | |
| --- | --- |
| Authors | Pengo et al. |
| Year of publication | 2018 |
| Country | United Kingdom |
| Study design | |
| Study conditions (*N*) | - UC (n=36, 2- and 6-week analyses conducted on subsample n=31 and n=25 respectively)  - UC + Positively framed messages (n=36, 2- and 6-week analyses conducted on subsample n=32 and n=31  respectively)  - UC + Negatively framed messages (n=37, 2- and 6-week analyses conducted on subsample n=31 and n=29  respectively) |
| Measurements | - Baseline  - 2 Weeks  - 6 Weeks |
| Study population | |
| Age (*M, SD*) | - 53.5 ± 12.5  - 46.7 ± 12.2  - 47.1 ± 11.7 |
| Gender (% female) | - 20.5  - 30.6  - 24.3 |
| Target population and recruitment strategy | Adults with OSAS starting CPAP treatment, recruited through sleep disorders centers at 2 hospitals. |
| Eligibility criteria | *Inclusion criteria:*  - Patients diagnosed with OSAS starting CPAP therapy  *Exclusion criteria:*  - Mental or physical disability precluding compliance with the protocol |
| Diagnostic procedure / OSA definition | Prospective screening using nocturnal pulse oximetry (Pulsox 300i, Konica Minolta Sensing Inc.,Tokyo, Japan) for two consecutive nights at home. Both a 4% oxygen desaturation index (4% ODI) ≥5 hour and typical symptoms of sleep apnea (Epworth Sleepiness Scale >10), or a 4% ODI greater than 15 hour were invited for CPAP |
| Interventions | |
| E-health condition |  |
| Add-on or replacement^1^ | Add-on |
| Type technology | Telephone |
| Type intervention | Usual care and follow-up care (see 'control condition') plus: Standardized motivational messages (either positively or negatively framed) were read out to patients during APAP collection appointment and through weekly telephone calls. Messages during appointment were read without adding any evaluative comments and without interruption. Calls lasted for 2-3 minutes and were non-interactive. The same messages were repeated once during weekly phone calls. Any other questions by the patients during these phone calls were directed to the clinical team (sleep specialists or qualified technicians) who phoned the patient back to address any clinical concerns. |
| Duration &  frequency | Motivational messages were read during clinic visit and weekly through telephone calls (+- 2 a 3 minutes) over period of 6 weeks |
| Control condition | Usual care and follow-up care:  Usual care consisted of 2 weeks APAP, 4 weeks CPAP. First, APAP collection session during which expert sleep technicians explained the importance of treating OSA and introduced APAP. Usual follow-up care furthermore included instructions on the use of their devices and one-to-one sessions were offered for patients experiencing difficulties. After 2 weeks, all patients were reviewed for troubleshooting and compliance assessment, and to exchange APAP for CPAP device. |
| Outcome(s) | |
| Assessment adherence | Automated data recorder in APAP device (S8/S9, ResMed Ltd, Sydney, Australia). Data was downloaded from SD cards. |
| Operationalization adherence | 2-week follow-up:  1) APAP use for >4 hours (% days)  2) APAP use for >4 hours (days)  3) APAP use for <4 hours (days)  4) APAP not used (days)  5) APAP average daily usage (hours)  6) APAP total hours used  6-week follow-up:  1) CPAP use for >4 hours (% days)  2) CPAP use for >4 hours (days)  3) CPAP use for <4 hours (days)  4) CPAP not used (days)  5) CPAP average daily usage (hours)  6) CPAP total hours used |
| Results | |
| Effects (*M, SD*) on adherence, incl. significance (*p*-values) | 2-Week follow-up:  1) Non-sign. (p=.06)  2) Non-sign. (p=.08)  3) Non-sign. (p=.99)  4) Non-sign. (p=.08)  5) Non-sign. (p=.10)  6) Sign. (p<.05): 40.8 ± 33.5 VS 53.7 ± 31.4 VS 35.6 ± 27.4  6-Week follow-up:  1) Non-sign. (p=.88): 50.1 ± 34.2 VS 45.7 ± 30.5 VS 46.1 ± 32.5  2) Non-sign. (p=.68): 21.1 ± 16.3 VS 19.2 ± 16.9 VS 19.7 ±17.7  3) Non-sign. (p=.39): 9.7 ± 8.9 VS 13.1 ± 9.0 VS 12.0 ± 9.0  4) Non-sign. (p=.88): 10.4 ± 12.0 VS 8.2 ± 8.4 VS 9.2 ±11.6  5) Non-sign. (p=.62): 3.1 ± 2.7 VS 3.5 ± 2.7 VS 2.6 ± 2.2  6) Non-sign. (p=.68): 132.8 ± 113.8 VS 1.9 ± 112.9 VS 118.5 ±97.4 |
| Other | |
| Source of funding and competing interest | The research was supported by the National Institute for Health Research (NIHR) Biomedical Research Center based at Guy’s and St. Thomas’ NHS Foundation Trust and King’s College London. |
| Study limitations and other comments | Missing 6-week data due to loss to follow-up, meaning that adherence data about the difference between groups should be interpreted with caution. |

| **Study reference #13** | |
| --- | --- |
| Authors | Sedkouaki et al. |
| Year of publication | 2015 |
| Country | France |
| Study design | |
| Study conditions (*N*) | - UC (n=190)  - UC + Telephone coaching (n=189) |
| Measurements | - Baseline  - 4 Months |
| Study population | |
| Age (*M, SD*) | - 60.8 ± 12.6  - 58.9 ± 13.7 |
| Gender (% female) | - 30.5  - 25.4 |
| Target population and recruitment strategy | Adults with SAHS starting CPAP treatment, recruited through multiple sites.  *Note: type of sites not specified* |
| Eligibility criteria | *Inclusion criteria:*  - Patients for clinical PSG evaluation who were subsequently diagnosed with SAHS and prescribed CPAP.  - Ability to understand and speak fluent French  - Able to complete the study questionnaires  *Exclusion criteria:*  - Aged <18 years  - Under guardianship  - Previous use of CPAP  - Psychiatric illness  - Participation in another clinical trial |
| Diagnostic procedure / OSA definition | Clinical PSG evaluation. Daytime sleepiness and > 3 of the following criteria: snoring, morning headaches, reduced attention, nocturia, AHT, decreased libido, associated with AHI ≥ 30/h. If AHI was below 30/h a PSG was performed and >10 arousals/h were required. |
| Interventions | |
| E-health condition |  |
| Add-on or replacement^1^ | Add-on |
| Type technology | Telephone |
| Type intervention | Usual follow-up care (see 'control condition') plus: Five educational telephone coaching sessions (day 3, 10, 30, 60, 90 with equipment at home) by competent staff. First session: assess patient’s knowledge about the disease, device and health consequences, and stress the importance of good adherence. Other educational telephone sessions focused on identifying disadvantages or obstacles to follow CPAP treatment, focusing on benefits linked to CPAP use, discussion of misconceptions about sleep apnea and barriers to use, concerns fears and beliefs, as well as the perceptions of their partners and family, in order to increase patients’ positive expectations regarding CPAP benefits. Any problems in links with SAHS encountered by the patient to the technician, psychologist or dietician (employed by the home care provider) were discussed. |
| Duration &  frequency | 5 phone calls of approximately 15-20 minutes over period of 3 months |
| Control condition | Usual follow-up care: home visit during first week of CPAP by technician delivering and re-explaining CPAP device and treatment. Further 1-month home follow-up visit by home care provider to check mask tolerance and functioning of machine, as well as 4-month visit to assess CPAP parameters. Sleep physician checked the compliance (patient questioning and machine data) and efficiency of CPAP treatment in month 1, 3, and 6. Finally, medical follow-up once a year. |
| Outcome(s) | |
| Assessment adherence | CPAP data registered by machine. |
| Operationalization adherence | 1) Primary outcome: % patients using CPAP for more than 3 hours per night for 4 months  2) Secondary outcome: mean nightly CPAP use in hours |
| Results | |
| Effects (*M, SD*) on adherence, incl. significance (*p*-values) | 1) Sign. (p-value not reported): 65.0 VS 75.0  2) Sign. (p=.04): 4.08+/−2.25 VS 4.34 +/− 2.17 |
| Other | |
| Source of funding and competing interest | Three authors have reported the following conflicts of interest: Nicole ROSSIN is employed by Sadir assistance, the home care provider. Ludivine LESEUX is employed by Sadir association. Mr. Jean-Louis FRAYSSE is the director of SADIR assistance. This study was funded by SADIR (home care provider). |
| Study limitations and other comments | Not all patients with coaching received the 5 phone calls as prescribed in the procedure due to business activity, holidays or patient requests to stop the phone calls. This might have biased results as a sign. and gradual link between patient phone calls received and mean hours of CPAP use was found. |

| **Study reference #14** | |
| --- | --- |
| Authors | Turino et al. |
| Year of publication | 2017 |
| Country | Spain |
| Study design | |
| Study conditions (*N*) | - UC (n=48)  - UC + Telemedicine care (n=52) |
| Measurements | - Baseline  - 1 Month  - 3 Months |
| Study population | |
| Age (*M, SD*) | - 54 ± 12  - 56 ± 13 |
| Gender (% female) | - 22.9  - 23.1 |
| Target population and recruitment strategy | Adults with OSA requiring CPAP treatment, recruited through sleep unit of university hospital. |
| Eligibility criteria | *Inclusion criteria:*  - Aged >18 years  - Newly diagnosed OSA  - Requiring treatment with CPAP (AHI >15 events/h)  *Exclusion criteria:*  - Impaired lung function (overlap syndrome, obesity hypoventilation and restrictive disorders)  - Severe heart failure  - Psychiatric disorders  - Periodic leg movements,  - Pregnancy  - Other dysomnias or parasomnias  - History of previous CPAP treatment |
| Diagnostic procedure / OSA definition | AHI >15 events/h. |
| Interventions | |
| E-health condition |  |
| Add-on or replacement^1^ | Add-on |
| Type technology | Telemonitoring, telephone |
| Type intervention | Usual follow-up care (see 'control condition') plus: Telemonitoring program collecting daily information about CPAP compliance, air leaks and residual respiratory events. Automatic alarms for the provider were generated in case of mask leaks (i.e. >30 L·min for >30% of the night) or usage of <4 hours a night on 2 consecutive nights. In case of alarm, the pulmonary specialist medical officer of the CPAP provider contacted the patient, providing case-by-case problem solving. This included suggestions about how to minimize symptoms (dry mouth, mask issues, discomfort with the device), specific interventions to improve compliance (mask changing, chin strap, pressure or humidifier settings, saline nasal sprays) and support for the patient in the use of CPAP. |
| Duration &  frequency | Daily telemonitoring and telephone calls as needed (see 'type intervention') over period of 3 months. |
| Control condition | Usual follow-up care: clinic visit after 1 month of CPAP treatment by the specialist nurse at the sleep unit for data gathering purposes. |
| Outcome(s) | |
| Assessment adherence | CPAP device (AirSense 10; ResMed, Martinsried, Germany) was equipped with mobile 2G (GSM/ GPRS) technology capable of sending daily information on CPAP adherence to a web database (i.e. MyOSA– Oxigen Salud; www.oxigensalud.com). |
| Operationalization adherence | Average nightly CPAP use in hours. |
| Results | |
| Effects (*M, SD*) on adherence, incl. significance (*p*-values) | 1-Month results: Non-sign. (p=.71): 5.2 ± 2.1 VS 4.8 ± 2.3  3-Month results: Non-sign. (p=.63): 4.9 ± 2.2 VS 5.1 ± 2.1 |
| Other | |
| Source of funding and competing interest | - |
| Study limitations and other comments | The high level of compliance in the standard management group could have masked any potential benefits of telemonitoring. |

| **Study reference #15** | |
| --- | --- |
| Authors | Fields et al. |
| Year of publication | 2016 |
| Country | Pennsylvania & New Jersey, US |
| Study design | |
| Study conditions (*N*) | - Traditional in-person care (n=28, analyses conducted on subsample n=20)  - Telemedicine care (n=32, analyses conducted on subsample n=14) |
| Measurements | - Baseline  - 3 Months |
| Study population | |
| Age (*M, SD*) | - 58.2 ± 14.4  - 46.7 ± 13.1 * |
| Gender (% female) | 6.0 |
| Target population and recruitment strategy | Adult veterans with OSAS starting +H17:I19APAP treatment, recruited through community-based outpatient centers. |
| Eligibility criteria | *Inclusion criteria:*  - Aged ≥ 18 years  - Received primary care at community-based outpatient center  - Fluent in English  - Diagnosis of OSA (AHI ≥ 5 events/h)  *Exclusion criteria:*  - Inability to return for follow-up sessions  - Previous diagnosis of sleep disordered breathing (OSA, central sleep apnea, Cheyne-Stokes respiration, obesity  hypoventilation syndrome) or narcolepsy. |
| Diagnostic procedure / OSA definition | AHI ≥ 15 events per hour or 5 ≤ AHI 5-15 events per hour determined by an overnight home sleep test, with clinical symptoms. |
| Interventions | |
| E-health condition |  |
| Add-on or replacement^1^ | Replacement |
| Type technology | Video conferencing, DVD, telephone |
| Type intervention | Visits and phone calls in according to standardized patient encounter template (i.e. similar to traditional in-person care). Initial clinical video tele-health visit, instructional DVD for home sleep testing, and 3 phone calls after starting APAP treatment: after 1 week (check start APAP treatment, encouragement, answer questions), as well as after 1 and 3 months (APAP unit data review, assessment of PAP-related concerns (e.g. mask leaks, claustrophobia), reinforcement, and opportunity for patients’ questions. |
| Duration &  frequency | Clinical-video tele-health visit of 40 minutes (at baseline), 1-week phone call of 10 minutes, 1- and 3-month phone calls of ≤20 minutes |
| Control condition | Visits and phone calls in according to standardized patient encounter template (i.e. similar to telemedicine care). Initial clinical in-person visit providing information about OSAS and CPAP treatment, 1-2 week return visit with instructions for home sleep testing unit for use that same night (i.e. no DVD), 1 phone call after starting APAP treatment (same brief encounter as the telemedicine arm), and in-person follow-up visits after 1 and 3 months. |
| Outcome(s) | |
| Assessment adherence | Wireless modem technology was used to transfer APAP adherence data from APAP units (Respironics  System One, Murrysville, PA) to EncoreAnywhere (Philips-Respironics), the latter being a HIPAA-compliant, password-protected internet database. |
| Operationalization adherence | 1) Average nightly APAP use in minutes  2) Average nightly APAP use in minutes on nights being used  3) % nights with device usage  4) % nights using device ≥ 4 hours |
| Results | |
| Effects (*M, SD*) on adherence, incl. significance (*p*-values) | 1) Non-sign. (p=.30): 175.6 ± 36.8 VS 220.8 ± 37.5  2) Non-sign. (p=.43): 268.9 ± 32.1 VS 305.7 ± 29.9  3) Non-sign. (p=.49): 54 ± 8 VS 65 ± 8  4) Non-sign. (p=.49): 39 ± 8 VS 47 ± 9 |
| Other | |
| Source of funding and competing interest | Financial support was provided by VISN 4 Competitive Pilot Project Fund. |
| Study limitations and other comments | Baseline difference in age. Relatively small sample size. Logistical considerations forced a lack of provider-site homogeneity: providers could not visit both recruitment sites or participate in both study arms. |

| **Study reference #16** | |
| --- | --- |
| Authors | Isetta et al. |
| Year of publication | 2015 |
| Country | Spain |
| Study design | |
| Study conditions (*N*) | - UC (n=70)  - Telemedicine-based CPAP follow-up (n=69) |
| Measurements | - Baseline  - 1 Month  - 3 Months  - 6 Months |
| Study population | |
| Age (*M, SD*) | - 49.0 ± 10.1  - 51.0 ± 8.9 *Note: Sign. difference, p-value not reported on* |
| Gender (% female) | 14.0 |
| Target population and recruitment strategy | Adults with OSA who were requiring CPAP treatment, recruited through 8 hospitals in Spain. |
| Eligibility criteria | *Inclusion criteria:*  - Diagnosis of OSA  - Requiring CPAP treatment  - Internet-connected device with a microphone and webcam  *Exclusion criteria:*  - Severe sleepiness  - Severe nasal obstruction  - Pregnancy  - Psychiatric disease  - Dangerous employment  - Clinical instability  - Current or previous treatment for OSA  - Lack of sufficient internet skills |
| Diagnostic procedure / OSA definition | Overnight sleep study which was scored by trained personnel, showing AHI / Respiratory Disturbance Index (RDI) ≥ 30. Or, AHI / RDI ≥ 5 and <30 plus symptoms related with sleep apnea-hypopnea syndrome and/or an Epworth Sleepiness Scale score ≥ 12 and/or associated comorbidity. |
| Interventions | |
| E-health condition |  |
| Add-on or replacement^1^ | Replacement |
| Type technology | Video-conferencing and web-based portal with education, self-monitoring and messaging tool. |
| Type intervention | CPAP follow-up care at distance:  1) video-conference follow-up visits (month 1 and 3)  2) extra tele-visits or telephone calls if needed  3) website with information about OSA and CPAP therapy, and a biweekly 6-item questionnaire about their  status, physical activity, sleep time, CPAP use and treatment side effects. Staff monitored questionnaire  answers and communicated with patients through the website messaging tool to solve treatment-related  problems.  *Note: Unclear whether low CPAP adherence fell under the above-mentioned ‘treatment-related problems’* |
| Duration &  frequency | Two follow-up video-conference visits (month 1 and 3), and extra tele-visits or hospitals visits as needed. |
| Control condition | Standard face-to-face follow-up care: hospital follow-up visits (month 1, 3, and 6), and extra visits or telephone calls if needed. |
| Outcome(s) | |
| Assessment adherence | n/a |
| Operationalization adherence | 1) Mean nightly CPAP use in hours  2) % adherers, defined as CPAP use >4 hours a night |
| Results | |
| Effects (*M, SD*) on adherence, incl. significance (*p*-values) | 6-Months results:  1) Non-sign. (p=.83): 4.2 ± 0.3 VS 4.4 ± 0.3  2) Non-sign. (p=.33): 57% VS 65% |
| Other | |
| Source of funding and competing interest | This project was supported by SEPAR/FIS PI14/00416 and ECO2013-47092 (MINECO, Spain) |
| Study limitations and other comments | Noteworthy: approximately 40% of screened patients lacked sufficient computer expertise/skills. |

| **Study reference #17** | |
| --- | --- |
| Authors | Stepnowsky et al. |
| Year of publication | 2007 |
| Country | California, United States |
| Study design | |
| Study conditions (*N*) | - UC (n=21, analyses conducted on subsample n=20)  - Telemonitored care (n=24, analyses conducted on subsample n=20) |
| Measurements | - Baseline  - 2 Months |
| Study population | |
| Age (*M, SD*) | 59 ± 14.3 |
| Gender (% female) | 0.2 |
| Target population and recruitment strategy | Adults with OSA starting CPAP treatment, recruited through referrals of physicians to sleep clinic by means of veterans affairs healthcare system. |
| Eligibility criteria | *Inclusion criteria:*  - Diagnosis of moderate-to-severe OSA (AHI ≥ 15 events per hour)  - Naive to CPAP therapy  - Stable sleep environment (operationally defined as a permanent address, requisite for wireless monitoring)  - Aged ≥ 18 years  *Exclusion criteria:*  - Allergies or sensitivity to the mask or mask material  - Previous use of any other PAP device (e.g., bi-level PAP, auto-adjusting PAP)  - Current use of prescribed supplemental oxygen  - Significant comorbid medical conditions that would prevent the patient from completing the protocol (i.e. any  medical or mental health condition that could interfere with the daily use of CPAP)  - When living in geographically unsuitable region (i.e., outside of the wireless network coverage area). |
| Diagnostic procedure / OSA definition | Sleep study demonstrating AHI ≥ 15 events/h. |
| Interventions | |
| E-health condition |  |
| Add-on or replacement^1^ | Replacement |
| Type technology | Telemonitoring |
| Type intervention | Daily telemonitoring (2-months) of compliance and efficacy data (mask leaks, AHI) and acting on those data collaboratively, and in partnership, with the patient. Collaborative management refers to the joint decision making and partnership between provider and patient and is characterized by communication, negotiation, and consideration of important patient factors and preferences. The frequency and nature of the clinical interactions depended on both the objectively measured nightly data values and subjective patient reports. Thresholds for the compliance and efficacy data: CPAP compliance (i.e. 4 hours a night), AHI (i.e. 10 events/hours of sleep), and mask leak (0.4 L/s). |
| Duration &  frequency | Daily telemonitoring and corresponding collaborative management over 2 months |
| Control condition | Usual follow-up care: 1-week telephone call after CPAP initiation and a 1-month in-office follow-up visit by CPAP clinic staff. Patients were encouraged to call the clinic any time they had a problem or concern. CPAP compliance and efficacy data were downloaded at the 1-month time point to help direct clinical management. |
| Outcome(s) | |
| Assessment adherence | Device-internal clock counter. CPAP device (AutoSet Spirit flow generator unit, ResMed Corp, Poway, CA) with attached ResTraxx wireless transmitter (ResMed Corp, Poway, CA). De-identified data were transmitted to a computer server. Research and clinical staff had secured access to data via standard browser and entry into the ResTraxx Data Center, the management website designed for 24/7 access to telemonitored data. |
| Operationalization adherence | 1) Mean nightly CPAP use in hours on all days  2) Mean nightly CPAP use in hours on nights being used  3) % nights in which CPAP was used >0 hours  4) % nights in which CPAP was used >4 hours |
| Results | |
| Effects (*M, SD*) on adherence, incl. significance (*p*-values) | 1) Non-sign. (p=.07): 2.8 ± 2.2 VS 4.1 ± 1.8  2) Non-sign. (p=.10): 3.8 ± 2.3 VS 5.0 ± 1.8  3) Non-sign. (p=.07): 60 ± 32 VS 78 ± 22  4) Non-sign. (p=.16): 37 ± 34 VS 52 ± 27 |
| Other | |
| Source of funding and competing interest | The study was supported in part by the VA San Diego Healthcare System, and the Veterans Medical Research Foundation. |
| Study limitations and other comments | Limited sample size to detect effects due to underestimated residual variance.  The study design dictated that both groups have ResTraxx wireless devices attached to their flow generator units. Given possible placebo effects, the UC group did not necessarily receive usual and customary care because of the presence of the wireless unit. Hence, this study may have  underestimated the effect of telemonitoring on CPAP compliance. |

| **Study reference #18** | |
| --- | --- |
| Authors | Stepnowsky et al. |
| Year of publication | 2013 |
| Country | California, United States |
| Study design | |
| Study conditions (*N*) | - UC (n=115)  - 'MyCPAP': Internet-based intervention based on wireless telemonitoring care (n=126)  *Note: 7 participants withdrew, but unclear from which study condition and how missing data were dealt with.* |
| Measurements | - Baseline  - 2 Months  - 4 Months |
| Study population | |
| Age (*M, SD*) | 52.1 ± 13.3 |
| Gender (% female) | n/a |
| Target population and recruitment strategy | Adults with OSA starting CPAP treatment, recruited through sleep medicine center of a university. |
| Eligibility criteria | *Inclusion criteria:*  - Diagnosis of OSA (AHI ≥ 15)  - CPAP therapy prescription  - Aged ≥ 18 years  *Exclusion criteria:*  - Residence in a geographical area outside of San Diego County (which could make regular contact and  participation difficult)  - Fatal comorbidity (life expectancy < 6 months as indicated by treating physician)  - Significant documented substance/chemical abuse. |
| Diagnostic procedure / OSA definition | AHI ≥ 15 events/h as measured by an overnight sleep study. |
| Interventions | |
| E-health condition |  |
| Add-on or replacement^1^ | Replacement |
| Type technology | Telemonitoring, web-based portal for education and self-monitoring |
| Type intervention | MyCPAP was comprised of as-needed clinical contacts, based on objectively measured CPAP adherence and efficacy data and access to a patient-oriented website. MyCPAP Website had the following main goals: (a) allow both the patient and provider access to telemonitored adherence and efficacy data on a daily basis (b) act on that data collaboratively to guide CPAP management and troubleshoot problems early and effectively, and (c) emphasize ways for the patient to express their preferences and needs. The website included  1) Education section providing basic education about sleep apnea, CPAP, and collaborative management  2) CPAP data section including easy-to-read charts that show CPAP adherence (in hours a night) and CPAP  efficacy data (disease severity as measured by number of apneas and hypopneas per hour) and amount of air  leak (in liters/min).  3) Graph section including both easy-to-complete individual items for patients to track, including sleepiness  levels and other patient-selected OSA-related symptoms.  4) Troubleshooting guide: interactive guide that allowed patients to select the CPAP problem they were having,  and possible causes and solutions were listed accordingly.  5) CPAP user’s manual including animations. |
| Duration &  frequency | CPAP telemonitoring every day throughout the active 2-month treatment period. |
| Control condition | Usual follow-up care: clinical contacts at predetermined times (1 week, 1 month) by CPAP clinic staff. Also, patients were encouraged to call whenever they had a problem or concern. Adjustments or changes in  the mask interface as well as pressure level changes were conducted if warranted. If the patient brought in their CPAP unit, the data was downloaded and utilized. |
| Outcome(s) | |
| Assessment adherence | UC participants: digital data smart card in CPAP device (PAP Autoset II, ResMed,San Diego,CA) recorded the amount of time the machine was used therapeutically. Data were downloaded from the smart card.  MyCPAP participants: wireless modem attached to PAP device, which could send data from device to Web-portal accessible by our team. The web-portal (“Restraxx Data Center,” or RDC), is comprised of the wireless module and the server/ database, which houses the data and restricts access to authorized health care professionals. |
| Operationalization adherence | Mean nightly CPAP use in hours. |
| Results | |
| Effects (*M, SD*) on adherence, incl. significance (*p*-values) | 2-Month results: Sign. (p=.02): 3.4 ± 2.4 VS 4.1 ± 2.3  4-Month results: Sign. (p=.03): 3.2 ± 2.4 VS 3.9 ± 2.3 |
| Other | |
| Source of funding and competing interest | This research was primarily supported by the AHRQ  1R18HS017426-01 and in part by the VA San Diego Research Service and Veteran’s Medical Research Foundation. |
| Study limitations and other comments | - |
| **Study reference #19** | |
| Authors | Taylor et al. |
| Year of publication | 2006 |
| Country | Washington, United States |
| Study design | |
| Study conditions (*N*) | - UC (n=58, analyses conducted on subsample n=49)  - Telemedicine care (n=56, analyses conducted on subsample n=47)  Note: analyses presumably conducted on subsample; method handling missing data unclear |
| Measurements | - Baseline  - 1 Month |
| Study population | |
| Age (*M, SD*) | - 44.6 ± 8.5  - 45.8 ± 10 |
| Gender (% female) | - 29.0  - 34.0 |
| Target population and recruitment strategy | Adults with OSAS starting CPAP treatment, recruited through university-affiliated sleep disorders center. |
| Eligibility criteria | *Inclusion criteria:*  - Patients diagnosed with OSAS starting CPAP therapy  *Exclusion criteria:*  - Current or previous treatment with nasal CPAP or other therapies such as an oral appliance or surgery for OSAS |
| Diagnostic procedure / OSA definition | A respiratory disturbance index (RDI) > 4 accompanied by symptoms of excessive daytime sleepiness. OSAS severity was determined by RDI events per hour: 5–14=mild, 15-29=moderate, and ≥30= severe. |
| Interventions | |
| E-health condition |  |
| Add-on or replacement^1^ | Replacement |
| Type technology | Tele-self-monitoring, automated computer-based support and feedback, telephone |
| Type intervention | Daily telemonitoring through self-report as provided via a home computer called the “Health Buddy". The Health Buddy OSAS Library was customized with information and suggested interventions. The library comprised preprogrammed questions and answers in a patient–provider dialogue covering four general aspects of OSAS care: symptom management, health behavior, knowledge, and general questions. Patient–provider dialogues were designed to provide education in the pathophysiology of OSAS, reinforce knowledge regarding nasal CPAP use, encourage skills mastery techniques and self-management behaviors, and interpret nasal CPAP symptoms and common side effects. High-risk patients (<4 hours of CPAP use during sleep for >3 days) were contacted by telephone by the sleep medicine practitioner within 24 hours. |
| Duration &  frequency | Daily self-monitoring and computer-based support and feedback, telephone calls in case of high risk of compliance only |
| Control condition | Usual follow-up care: Clinic visit 1 month after starting CPAP and any subsequent clinic visits felt necessary by the care provider. Also, participants were able to access the sleep medicine practitioner for telephone consultations and walk-in visits. |
| Outcome(s) | |
| Assessment adherence | Automated data recorder in the CPAP device |
| Operationalization adherence | 1) Average nightly CPAP use in hours over 30-day observation period  2) % nights CPAP use of ≥ 4 hours on all of the nights monitored over 30-day observation period |
| Results | |
| Effects (*M, SD*) on adherence, incl. significance (*p*-values) | 1) Non-sign. (p=.87): 4.22 ± 2.05 VS 4.29 ± 2.15  2) Non-sign. (p=.61): 50.1 ± 33.8 VS 46.9 ± 34.2" |
| Other | |
| Source of funding and competing interest | Supported by the Telemedicine Directorate, Walter Reed Army Medical Center, Washington, DC. |
| Study limitations and other comments | A confounding factor that may have blunted a difference between the randomized groups was the similar availability of follow-up care to each group. Such follow-up care included telephone contact and walk-in access to care. The increased number of telephonic contacts to the telemedicine group was balanced by walk-in care accessed by the traditional care group. Within the telemedicine group, 7 of the 9 participant who withdrew during study, did so because of failure to activate the Health Buddy computer. |

UC = Usual care; CG = Control group; OSA = obstructive sleep apnea; OSAS = obstructive sleep apnea syndrome; OSAHS = Obstructive Sleep Apnea Hypopnea Syndrome; SAHS = Sleep Apnea/Hypopnea Syndrome; CPAP=Continuous positive airway pressure; APAP=Automatically-Adjusting Positive Airway Pressure; PAP: Positive Airway Pressure; AHI = Apnea/Hypopnea Index; Sign. = Significant; PSG = polysomnography

^1^ Indicates whether the E-health intervention was an add-on, or replacement of usual (follow-up) care as provided during treatment of CPAP in the control condition.

* p < .05
